# Alterations in the Abundance and Co-occurrence of *Akkermansia muciniphila* and *Faecalibacterium prausnitzii* in the Colonic Mucosa of Inflammatory Bowel Disease Subjects

**DOI:** 10.3389/fcimb.2018.00281

**Published:** 2018-09-07

**Authors:** Mireia Lopez-Siles, Núria Enrich-Capó, Xavier Aldeguer, Miriam Sabat-Mir, Sylvia H. Duncan, L. Jesús Garcia-Gil, Margarita Martinez-Medina

**Affiliations:** ^1^Laboratory of Molecular Microbiology, Biology Department, Universitat de Girona, Girona, Spain; ^2^Department of Gastroenterology, Hospital Dr. Josep Trueta, Girona, Spain; ^3^Department of Gastroenterology, Hospital Santa Caterina, Girona, Spain; ^4^Microbiology Group, Rowett Institute of Nutrition and Health, Aberdeen, United Kingdom

**Keywords:** *Akkermansia muciniphila*, *Faecalibacterium prausnitzii*, Crohn's disease, ulcerative colitis, inflammatory bowel diseases

## Abstract

*Akkermansia muciniphila* and *Faecalibacterium prausnitzii*, cohabitants in the intestinal mucosa, are considered members of a healthy microbiota and reduction of both species occurs in several intestinal disorders, including inflammatory bowel disease. Little is known however about a possible link between the reduction in quantity of these species, and in which circumstances this may occur. This study aims to determine the abundances and co-occurrence of the two species in order to elucidate conditions that may compromise their presence in the gut. Loads of *A. muciniphila*, total *F. prausnitzii* and its two phylogroup (16S rRNA gene copies) were determined by quantitative polymerase chain reaction in colonic biopsies from 17 healthy controls (H), 23 patients with ulcerative colitis (UC), 31 patients with Crohn's disease (CD), 3 with irritable bowel syndrome (IBS) and 3 with colorectal cancer (CRC). Data were normalized to total bacterial 16S rRNA gene copies in the same sample. Prevalence, relative abundances and correlation analyses were performed according to type of disease and considering relevant clinical characteristics of patients such as IBD location, age of disease onset, CD behavior, current medication and activity status. Co-occurrence of both species was found in 29% of H, 65% of UC and 29% of CD. Lower levels of total *F. prausnitzii* and phylogroups were found in subjects with CD, compared with H subjects (*P* ≤ 0.044). In contrast, no differences were found with the regard to *A. muciniphila* abundance across different disease states, but CD patients with disease onset below 16 years of age featured a marked depletion of this species. In CD patients, correlation between *A. muciniphila* and total *F. prausnitzii* (ρ = 0.362, *P* = 0.045) was observed, and particularly in those with non-stricturing, non-penetrating disease behavior and under moderate immunosuppressants therapy. Altogether, this study revealed that co-occurrence of both species differs between disease status. In addition, IBD patients featured a reduction of *F. prausnitzii* but similar loads of *A. muciniphila* when compared to H subjects, with the exception of those with early onset CD. Depletion of *A. muciniphila* in this subgroup of subjects suggests that it could be a potential biomarker to assist in pediatric CD diagnosis.

## Introduction

Crohn's disease (CD) and ulcerative colitis (UC) are the two major types of idiopathic inflammatory bowel diseases (IBD) (Mendoza Hernández et al., 2007). Both are chronic inflammatory disorders of the gut. UC typically begins in the rectum and inflammation may extend continuously to involve the entire colon. In 20% of CD patients the disease affects the colon exclusively (Silverberg et al., 2005), but the most commonly involved areas are terminal ileum and the beginning of the colon. In CD any part of the gastrointestinal tract (from the oropharynx to the anus) may be affected in a patchy pattern (Mendoza Hernández et al., 2007). Other than location, differences in the mucosal lesions exist between these conditions. Inflammation in CD can be transmural reaching the serosa, whereas inflammation in UC patients is generally restricted to the mucosa.

The inner layer of the bowel wall is a niche of particular importance, because of the spatial proximity between epithelial cells and gut bacteria, and thus the study of human intestinal mucosa biopsies provides meaningful insights of host-bacterial interactions. Numerous studies have been prompted over the last decade aiming at deciphering the exact role of gut microbiota in IBD. Nowadays there is a wide variety of clinical and experimental studies revealing microbial implication in IBD (Sartor, 2006, 2008; Seksik et al., 2006; Manichanh et al., 2012). The most replicated finding by far has been disturbances in the intestinal microbiota composition balance, situation known as dysbiosis (Gophna et al., 2006; Manichanh et al., 2006; Martinez-Medina et al., 2006; Andoh et al., 2009; Willing et al., 2009; Sokol and Seksik, 2010; Joossens et al., 2011; Mondot et al., 2011; Machiels et al., 2013). In this state, dominating species, to whom a beneficial role to preserve gut homeostasis has been attributed, become underrepresented.

*Akkermansia muciniphila* inhabits mainly in the mucosa, and represents between 1 and 3% of the gut microbiota (Derrien et al., 2004, 2008). A decrease of this species has been demonstrated in feces and/or biopsies of several disorders including autism, obesity, type 2 diabetes, appendicitis, and IBD (Belzer and de Vos, 2012; Everard et al., 2013). Studies in mice models have shown that gut colonization by this species affects expression of genes involved in immune response-regulatory processes (Derrien et al., 2011) as well as in host's lipid metabolism (Lukovac et al., 2014), especially in the colon. It is of note that extracellular vesicles derived from this species have a protective function that ameliorates severity of induced colitis in mice, suggesting that it has an important role in the maintenance of intestinal homeostasis (Kang et al., 2013). *A. muciniphila* is essential for a healthy mucus layer in the human gut in terms of mucus production and thickness (Belzer and de Vos, 2012). This species is not only important for the host, but also for gut microbial community. Its specific capability to degrade mucus results in the release of oligosaccharides and the production of propionate and acetate (Derrien et al., 2004) as well as amino acids, important co-factors and vitamins (van Passel et al., 2011) that become available for other gut symbionts. However, significant co-occurrence of this species with other bacterial taxa present in the gut has not been revealed in feces (Lozupone et al., 2012).

In turn, *Faecalibacterium prausnitzii* is also an abundant intestinal microorganism with a feco-mucosal distribution, and whose relative abundance can represent between 2 and 15% of intestinal bacterial communities (Swidsinski et al., 2005; Baumgart et al., 2007; Flint et al., 2012). Several studies, of fecal and/or mucosal samples, have shown that *F. prausnitzii* prevalence and abundance are reduced under certain disorders such as celiac disease (Swidsinski et al., 2008; De Palma et al., 2009), obesity and type 2 diabetes (Furet et al., 2010; Graessler et al., 2012), appendicitis (Swidsinski et al., 2011), chronic diarrhea (Dörffel et al., 2012), irritable bowel syndrome (IBS) of alternating type (Rajilić-Stojanović et al., 2011), colorectal cancer (CRC) (Balamurugan et al., 2008; Lopez-Siles et al., 2016), and particularly in IBD (Sokol et al., 2008, 2009; Swidsinski et al., 2008; Willing et al., 2009; Machiels et al., 2013; Lopez-Siles et al., 2014). Low abundance of this species has been linked with active IBD (Sokol et al., 2009), and some complications such as a higher risk of post-operative recurrence (Sokol et al., 2008) or pouchitis (McLaughlin et al., 2010). Other than butyrate production (which can reduce intestinal mucosa inflammation and is the main energy source for the colonocytes), additional anti-inflammatory properties have been attributed to *F. prausnitzii* (Sokol et al., 2008; Miquel et al., 2013; Martín et al., 2014). Both, cell and supernatant fractions of this species, have been proven to reduce severity of acute (Sokol et al., 2008; Rossi et al., 2015), chronic (Martín et al., 2014) and low grade (Martín et al., 2015) inflammation in murine models. This has been attributed to an enhancement of intestinal barrier function related with the expression of certain tight junction proteins other than claudin (Carlsson et al., 2013). *F. prausnitzii* also influences gut physiology through the production of mucus O-glycans, and may help to maintain suitable proportions of different cell types of secretory linage in the intestinal epithelium, as evidenced in rodent studies (Wrzosek et al., 2013). To date, it remains unclear which conditions are likely to compromise this species in the gut. Alterations in gut pH or bile salt concentration have been suggested (Lopez-Siles et al., 2012), but a break in the ecologic relations with other gut symbionts that support its presence in the gut may also contribute, but have been little studied. Co-occurrence network analysis of gut bacteria found in feces, showed that *F. prausnitzii* co-occurs with several members of the *C. coccoides* group and Bacteroidetes (Lozupone et al., 2012). As *F. prausnitzii* growth is stimulated by acetate (Duncan et al., 2002), its presence in the gut may also be favored by acetate producers like *A. muciniphila*. However, little is known about interaction between these two species.

This work is aimed at determining the variation of mucosa-associated *A. muciniphila* and *F. prausnitzii* between healthy control subjects (H) and patients suffering from IBD, in order to elucidate in which conditions imbalances of these species take place, and if both species are affected equally. Some IBS and CRC patients have been included for comparative purposes. Prevalence and abundance of mucosa-associated *A. muciniphila* and *F. prausnitzii* have been determined in colonic samples by quantitative polymerase chain reaction (qPCR). Data have been analyzed taking into account patients' most relevant clinical characteristics. Medication at sampling was also considered in order to determine if any of the current therapies are effective in restoring these species levels to those found in H. In addition, correlation analysis of their load has also been conducted to provide supporting evidence on the effect of one population over the other, or about whether or not they are influenced by similar gut factors.

## Materials and methods

### Patient recruitment and characteristics

The study population was a cohort consisting of Spanish volunteers including 54 IBD (31 CD and 23 UC), three IBS, three CRC patients and 17 H (Table 1). Subjects were gender matched for all the groups. Concerning age, CD patients were younger than those in the H group (*P* = 0.002), whereas CRC patients were significantly older than those with IBD (*P* ≤ 0.028). Besides, at disease onset, CD patients were younger than UC (mean age ± SD; UC = 37.2 ± 13.3 years, CD = 28.0 ± 12.4 years; *P* = 0.012).

**Table 1:** Sample size and clinical characteristics of subjects.

	**Healthy**	**Irritable bowel syndrome**	**Colorectal cancer**	**IBD**		***P*-value^§^**
				**Ulcerative colitis**	**Crohn's disease**	
*n* (patients)	17	3	3	23	31	
Age (mean years ± SD)	48.1 ± 15.8	35.0 ± 7.0	69.3 ± 16.0	40.5 ± 13.5	33.6 ± 12.2	0.002^**^
Male (*n*, %)	10 (58.8%)	1 (33.3%)	3 (100%)	13 (56.5%)	20 (64.5%)	0.826^†^
Active (*n*, %)	na	na	na	3 (13.0%)	13 (41.9%)	**0.036**^†^
Previous surgery (*n*, %)	na	na	na	1 (4.3%)	6 (19.4%)	0.213^†^
Smokers (*n*, %)	0 (0%)	0 (0%)	0 (0%)	2 (8.7%)	5 (16.1%)	0.410^†^
Treatment (*n*, %)^*^						0.494^†^
No treatment	na	na	na	11 (47.8%)	10 (32.3%)	
Mesalazine	na	na	na	3 (13.0%)	3 (9.7%)	
Moderate immunosuppressant	na	na	na	4 (17.4%)	10 (32.3%)	
Anti-TNFα (infliximab, adalimumab)	na	na	na	3 (13.0%)	6 (19.4%)	
CD Montreal classification						
Age of diagnosis (*n*, %)^*^						**0.007**
<16 yeays (A1)	na	na	na	na	5 (16.1%)	
17–40 years (A2)	na	na	na	11 (47.8%)	21 (67.7%)	
>41 years (A3)	na	na	na	9 (39.1%)	3 (9.7%)	
Location (*N*, %)						na
Ileal-CD (L1)	na	na	na	na	13 (41.9%)	
Colonic-CD (L2)	na	na	na	na	7 (22.6%)	
Ileocolonic-CD (L3)	na	na	na	na	10 (32.3%)	
Behavior (*N*, %)^*^						na
Non-stricturing, non-penetrating (B1)	na	na	na	na	19 (61.3%)	
Stricturing (B2)	na	na	na	na	8 (25.8%)	
UC classification (*n*, %)^*^						na
Ulcerative proctitis (E1)	na	na	na	5 (21.7%)	na	
Distal UC (E2)	na	na	na	11 (47.8%)	na	
Extensive UC or ulcerative pancolitis (E3)	na	na	na	6 (26.1%)	na	

IBD, Inflammatory bowel disease; TNF, tumor necrosis factor; na, not applicable.

* Medical treatment at the time of sampling was available in 21/23 UC, and 29/31 CD patients; Age of disease onset was available for 20/23 UC patients, and 29/31 CD patients; Disease behavior at last follow-up before the time of sampling was available in 27/31 CD patients, and none had penetrating CD (B3); Maximal disease extent at the time of sampling was available in 22/23 UC and 30/31 CD patients.

§ Groups were compared by appropriate statistical tests, and P-value ≤ 0.05 was considered significant, χ^2^ test,

** *ANOVA. Analyses statistically significant are highlighted in boldface*.

Subjects were recruited by the Gastroenterology Services of the Hospital Universitari Dr. Josep Trueta (Girona, Spain) and the Hospital Santa Caterina (Salt, Spain) between 2006 and 2010. To avoid bias between centers, patients with IBD were diagnosed according to standard clinical, pathological, and endoscopic criteria and categorized according to the Montreal classification (Silverberg et al., 2005). IBS patients were diagnosed according to Rome III criteria (available at http://www.romecriteria.org/criteria/). The diagnosis of CRC was established by colonoscopy and biopsy, and data correlated with high risk of developing this disease was recorded. Controls consisted of subjects who underwent colonoscopy for different reasons as rectorrhagia (*n* = 8), colorectal cancer familial history (*n* = 3), and abdominal pain (*n* = 6), and all featured normal colonoscopy. Clinically relevant data of all the patients was collected (Table 1). Percentage of active patients was higher in CD patients than in UC (*P* = 0.036). Individuals included in this study were >18 years old, did not have any other intestinal disease and were not pregnant. Antibiotic treatment within 2 months before colonoscopy was an exclusion criterion.

### Ethics statement

This work was approved by the Ethics Committee of Clinical Research of the Hospital Universitari Dr. Josep Trueta (Girona, Spain) and the Institut d'Assistència Sanitària of Girona (Salt, Spain) on 26th May 2006 (protocol code BACTECCU, Ref CEIC: 08/06) and 21st April 2009 (protocol code BACTODIAG, Ref CEIC: 10/08), respectively. All subjects gave written informed consent in accordance with the Declaration of Helsinki.

### Sample collection and DNA extraction

Cleansing of the gastrointestinal tract using Casenglicol® was performed prior to colonoscopy, following manufacturer's guidelines. During routine endoscopy and following standard procedures, a biopsy (<25 mg) from non-affected tissue of the colon was taken for each subject. All biopsies were immediately placed in sterile tubes without any buffer. Following completion of the whole endoscopic procedure, all samples were stored at −80°C upon analysis.

To discard transient and loosely attached bacteria, biopsies were subjected to two mild ultrasound wash cycles, as reported previously (Martinez-Medina et al., 2006). Afterwards, DNA was extracted using the NucleoSpin® Tissue Kit (Macherey-Nagel GmbH & Co., Duren, Germany). The support protocol for Gram positive bacteria (consisting of pre-incubation during 1 h at 37°C with buffer T1 (20 mm Tris/HCl, 2 mM EDTA, 1% Triton X-100, pH 8) supplemented with 20 mg/ml lysozyme), and the RNAse treatment step were carried out. Genomic DNA was eluted with 10 mM Tris-HCl (pH 7.4) and stored at −80°C until use. DNA concentration and purity of the extracts were determined with a NanoDrop ND-100 spectrophotometer (NanoDrop Technologies, USA). The average purity and concentration of the DNA extracts was (mean ± SD) 1.794 ± 0.579 Abs260/280 ratio and 174.864 ± 131.829 ng/μl, respectively.

### Quantification standards for qPCR

Standard DNA templates from *F. prausnitzii* strain S3L/3 (phylogroup I), *F. prausnitzii* DSM 17677 (phylogroup II) and *A. muciniphila* (ATCCBAA-835) were prepared as genetic constructs after PCR amplification of the whole 16S rRNA as previously reported (Lane, 1991; Weisburg et al., 1991), and subsequent insertion of this gene into a pCR®4-TOPO® cloning plasmid (Invitrogen, CA, USA) following manufacturer's guidelines.

After purification with the NucleoSpin® Plasmid kit (Macherey-Nagel GmbH & Co., Duren, Germany), plasmids were linearized with *SpeI* and quantified using Qubit™ Quantitation Platform (Invitrogen, Carlsbad, USA). Initial target concentration was inferred taking into consideration the theoretical molecular weight (3.58 × 10^6^ Da) and the size of recombinant plasmid (5421 pb).

Standard curves were obtained from 10-fold serial dilutions of the titrated suspension of linearized plasmids. Strains used to construct each standard curve are indicated in Table 2. To prepare the standard curve, only dilutions within the linear dynamic range span of each reaction were used, as detailed in Table 2. Total bacteria 16S rRNA gene quantification was used to intercalibrate all the standard curves, in order to make sure that results obtained were comparable. Accordingly, plasmid preparations for *A. muciniphila* standard and phylogroups standards were run as unknown samples in a total bacterial qPCR. Specific intercalibration of total *F. prausnitzii* qPCR was not required as it uses the same standard curve template employed for total bacterial qPCR (Table 2). Quantification values obtained were compared to initial target concentration inferred from DNA concentration, and <10% variation was obtained.

**Table 2:** 16S rRNA-targeted primers and probes used in this study.

**Target**	**Primers and probe**	**Sequence (5′-3′)^a^**	**Final conc. (nM)**	**Strain used as standard**	Standard curve^*^	**qPCR conditions**^c^					**References**
						**Fluorescence reporting method**	**Cycles**	**Denat. T^a^(°C); *t* (s)**	**Annealing and extension T^a^(°C), *t* (s)**	**Melting curve^d^**
Total bacteria (Eubacteria)	F_Bact 1369	CGGTGAATACGTTCCCGG	300	*F. prausnitzii* DSM 17677	10^7^-10^3^; 89.0 ± 7.0	Hydrolysis probe	40	95; 30	60; 60	No	Furet et al., 2009
	R_Prok1492	TACGGCTACCTTGTTACGACTT	300								
	**P_TM1389F**	**FAM-CTTGTACACACCGCCCGTC-TAMRA**	**250**								
*Akkermansia muciniphila*	AM1-F	CAGCACGTGAAGGTGGGGAC	300	*A. muciniphila* ATCCBAA-835	2 × 10^2^ - 2 × 10^7^; 93.9 ± 4.2	SYBR Green	50	95; 15	65; 30 and 72; 32	Yes	Collado et al., 2007
	AM2-R	CCTTGCGGTTGGCTTCAGAT	300								
*Faecalibacterium prausnitzii* (total)	Fpra428F	TGTAAACTCCTGTTGTTGAGGAAGATAA	300	*F. prausnitzii* DSM 17677	10^7^-10^3^; 84.8 ± 3.2	Hydrolysis probe	40	95; 15	60; 60	No	Lopez-Siles et al., 2014
	Fpra583R	GCGCTCCCTTTACACCCA	300								
	**Fpra493PR**	**FAM-CAAGGAAGTGACGGCTAACTACGTGCCAG-TAMRA**	**250**								
IAC^b^	IAC F	TACGGATGAGGAGGACAAAGGA	300	DNA IAC	n.a.	Hydrolysis probe	40	95; 15	60; 60	No	Lopez-Siles et al., 2014
	IAC R	CACTTCGCTCTGATCCATTGG	300								
	**IAC PR**	**VIC**®**-CGCCGCTATGGGCATCGCA-TAMRA**	**250**								
*F. prausnitzii* (phylogroups)	Fpra 136F	CTCAAAGAGGGGGACAACAGTT	900	*F. prausnitzii* S3L/3 (phylogroup I) and DSM 17677 (phylogroup II)	10^6^-10; 86.3 ± 6.1; 95.3 ± 11.9	Hydrolysis probe	40	95; 15	64; 60	No	Lopez-Siles et al., 2016
	Fpra 232R	GCCATCTCAAAGCGGATTG	900								
	**PHG1 180PR**	**6FAM-TAAGCCCACGACCCGGCATCG-BHQ1**	**300**								
	**PHG2 180PR**	**JOE-TAAGCCCACRGCTCGGCATC-BHQ1**	**300**								

* Range span (16S rRNA gene copies), Efficiency (mean ± standard deviation); n.a., not applicable.

a Probe sequences are in bold. FAM™, 6-carboxyfluorescin; VIC®, 6-carboxyrhodamine; JOE, 4′,5′-dichloro-2′,7′-dimethoxy-5(6)-carboxyfluorescein; TAMRA™, tetramethylrhodamin; BHQ1, Black Hole Quencher1.

b IAC, internal amplification control; 10^3^ copies of appropriate DNA template were added in each reaction. DNA IAC sequence (5′-3′), DNA IAC sequence: 5′-TACggATgAggAggACAAAggACgCCgCTATgggCATCgCACCAATggATCAgAgCgAAgTg-3′ (according to Lopez-Siles et al., 2014).

c An amperase treatment (50°C, 2 min) and an initial denaturing step (95°C, 10 min) were performed for all the reactions. For assays based on hydrolysis probes, annealing and extension steps were performed simultaneously.

d *Melting curve consisted on 95°C 15 s, 60°C 1 min, 95°C 15 s, and 60°C 15 s (average temperature slope 0.58°C/s)*.

### qPCR assays

Previously reported 16S rRNA gene-targeted primers and probes were used for total *F. prausnitzii* (Lopez-Siles et al., 2014), phylogroups (Lopez-Siles et al., 2016), *A. muciniphila* (Collado et al., 2007) and total bacterial (Furet et al., 2009) quantification through qPCR.

Amplification reactions were performed as described elsewhere (Collado et al., 2007; Furet et al., 2009; Lopez-Siles et al., 2014, 2016) with slight modifications detailed in Table 2. Briefly, quantifications were carried out in a total volume of 20 μl reactions containing: 1 × TaqMan® Universal PCR Master Mix 2 × or SYBR®Green PCR Master Mix 2 × (Applied Biosystems, Foster City, CA, USA) as required, 300–900 nM of each primer and 250–300 nM of probe if necessary. Up to 50 ng of genomic DNA template was added in each reaction. All primers and probes used in this study as well as PCR conditions are detailed in Table 2. Total *F. prausnitzii*, and total bacteria primers and hydrolysis probes were purchased from Applied Biosystems (Foster City, CA, USA), whereas primers and hydrolysis probes for *F. prausnitzii* phylogroups and *A. muciniphila* were acquired from Biomers (Ulm, Germany). DNA of the internal amplification control (IAC) was synthesized by Bonsai technologies group (Alcobendas, Spain). All oligonucleotides were purified by HPLC. Plates and optical caps were provided by Applied Biosystems (Ref. 4323032 and Ref. 4306737, respectively).

Samples were run at least in duplicate in the same plate (Table S1), which was set up manually. For data analysis, the mean of the quantifications was used. Duplicates were considered valid if the standard deviation between quantification cycles (C_q_) was <0.34 (i.e., a difference of <10% of the quantity was tolerated), and if not quantification was repeated. Quantification controls consisting of at least five reactions with a known number of target genes were performed to assess inter-run reproducibility. Inhibition was controlled on total *F. prausnitzii* quantification by adding 10^3^ copies of an internal amplification control (IAC) template to each reaction. It was considered that there was no inhibition if the obtained C_q_ was <0.34 different from those obtained when quantifying the IAC alone for any of the replicates. In each run, a non-amplification control (NTC) which did not contain any DNA template (either bacterial or IAC) was also included. In all cases with hydrolysis probes, NTC resulted in undetectable C_q_ values whereas for SYBR Green assays NTC had C_q_ >35, and melting curve analysis confirmed no specific amplification.

A 7500 Real Time PCR system (Applied Biosystems, USA) was used to perform all qPCR. The thermal profile used for each assay is detailed in Table 2. In summary, it consisted of a first step at 50°C during 2 min for amperase treatment followed by a 95°C hold for 10 min to denature DNA and activate Ampli-Taq Gold polymerase; and a further 40–50 cycles consisting of a denaturation step at 95°C for 15 s, followed by an annealing and extension step at 60°C (or at 64°C for phylogroups quantification) for 1 min. When required, melting curve analysis was performed to assess whether or not fluorescence was due to specific amplification products. Data were collected and analyzed using the 7500 SDS system software version 1.4 (Applied Biosystems). Assays were performed under average PCR efficiencies of (mean ± SD) 89.9 ± 4.6% (Figure S2).

### Data normalization and statistical analysis

Regarding the qualitative analyses, absence of *F. prausnitzii*, its phylogroups or *A. muciniphila* was considered if no detection was obtained during the qPCR analysis, corresponding to samples that carried these bacteria below the detection limit (i.e., 106.6, 1.10, 2.39, and 374.09 16S rRNA genes per reaction for total *F. prausnitzii*, phylogroup I, phylogroup II, and *A. muciniphila*, respectively). Pearson's χ^2^ test was used to compare the prevalence between groups of patients, by IBD disease location, age of disease onset and other clinically relevant data as activity, treatment and whether or not patients have had intestinal resection.

Referring to quantitative analyses, total *F. prausnitzii*, phylogroups and *A. muciniphila* 16S rRNA gene copy detected in each sample were normalized to the total bacterial 16S rRNA gene copies in the same sample. Data are given as the log_10_ of the ratio between 16S rRNA gene copies of the target microorganism and million of total bacterial 16S rRNA genes detected in the same sample. No further correction to adjust for differences in 16S rRNA gene operons in each species was performed, as no consensus has been achieved to date for *F. prausnitzii* according to *rrn*DB (Stoddard et al., 2015). For those samples with no detection of *F. prausnitzii*, phylogroup or *A. muciniphila*, the number of copies corresponding to their respective detection limit was used for calculations of relative abundances. Kruskal–Wallis non-parametric test was applied to assess differences in variables with more than two categories such as diagnostics, CD and UC disease location, age of disease onset, and current medication. Mann–Whitney *U* test was used to perform pairwise comparisons of subcategories of these variables, and FDR after multiple comparisons was assessed (Table S2). Mann–Whitney *U* test was also used to compare, variables with two categories such as activity (active CD and UC patients when CDAI > 150 (Best et al., 1976) and a Mayo score >3 (Pineton de Chambrun et al., 2010), respectively), and intestinal resection. In addition, ratios between 16S rRNA gene copies of either total *F. prausnitzii*, phylogroup I or phylogroup II and *A. muciniphila* were calculated and analyzed as detailed above. Spearman correlation coefficient and significance between total *F. prausnitzii*, or phylogroup quantities and *A. muciniphila* load was calculated. The same statistical method was used to analyze the correlation between the quantity of each bacterial group, and continuous clinical data such as time (in years) since disease onset. All the statistical analyses were performed using the SPSS 15.0 statistical package (LEAD Technologies, Inc.). Significance levels were established for *P* ≤ 0.05.

## Results

### Prevalence of mucosa-associated *A. muciniphila* and *F. prausnitzii*

**Figure 1:**
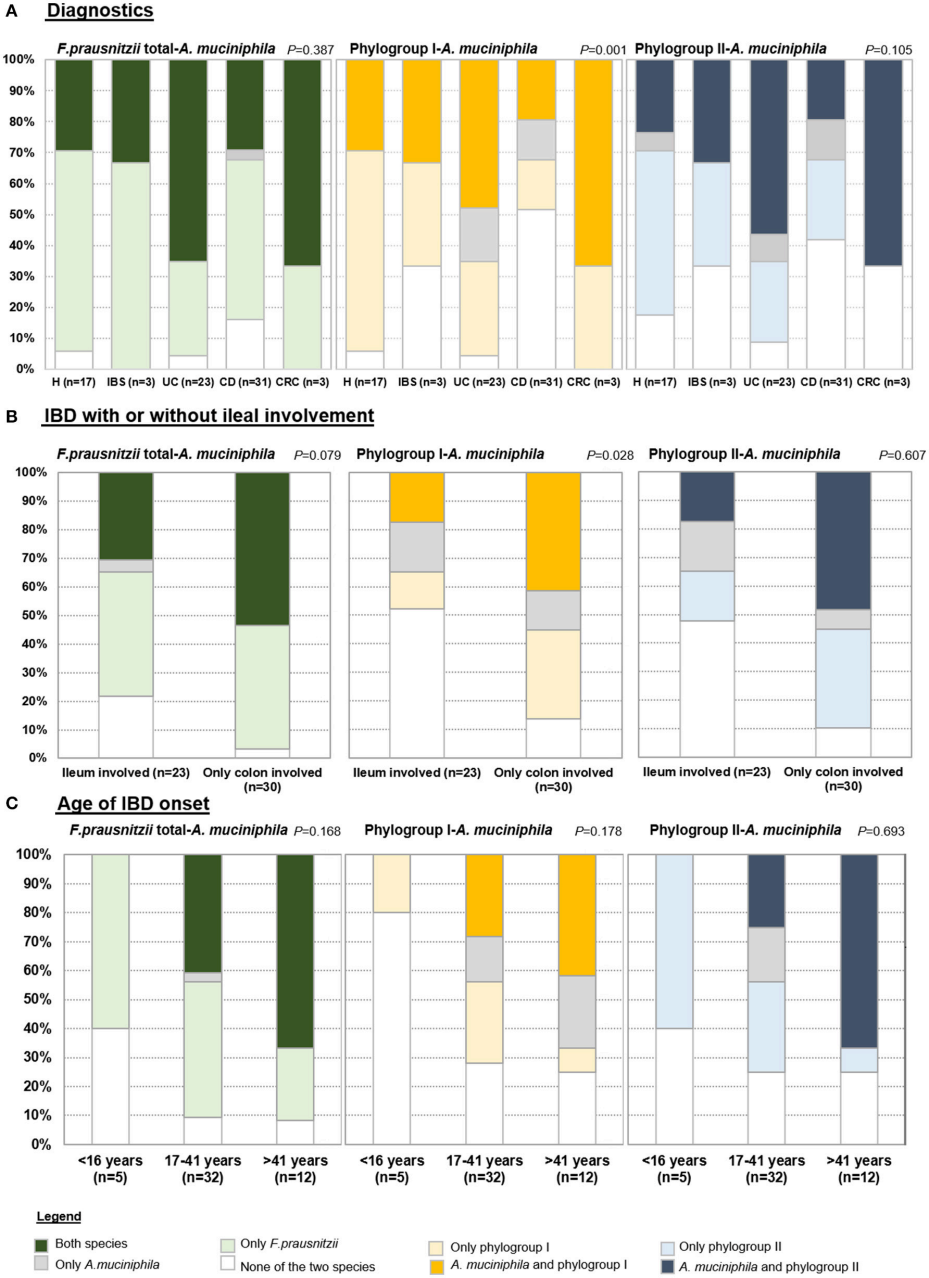
Prevalence of total *F. prausnitzii*, phylogroups and *A. muciniphila* in **(A)** each group of patients, **(B)** in Inflammatory bowel disease (IBD) subjects according to whether or not ileum is affected, and **(C)** in IBD by age of disease onset. H, control subjects; IBS, irritable bowel syndrome; UC, ulcerative colitis; CD, Crohn's disease; CRC, colorectal cancer.

To assess co-occurrence of both species, prevalence of *F. prausnitzii* (total or separating by phylogroups), and *A. muciniphila* as calculated from positive determinations over total samples, was analyzed by condition, by IBD location and also taking into account relevant clinical data (Figure 1). Four categories of patients were established based on: detecting only *F. prausnitzii*, detecting only *A. muciniphila*, detecting both species, or none of them. Most of the subjects carried both species or *F. prausnitzii* alone, whilst finding *A. muciniphila* alone was rare and in some cases, none of the two species was found suggesting that if present, they are below the detection limit of our assays.

When analysing the cohort by diagnostics (Figure 1A), only statistically significant differences in the proportions of each category were observed for the co-occurrence of *F. prausnitzii* phylogroup I and *A. muciniphila* (*P* < 0.001). As a particularity of CD, none of the two species was detected in 51.61% of subjects of this group, which was a higher proportion than in the other groups of subjects (ranging from 4.35% to 33.3%). In 64.71% of H subjects only one species (*F. prausnitzii* phylopgroup I) was detected, while this category was present in between 16% and 34% in the other groups of subjects. *F. prausnitzii* phylogroup I and *A. muciniphila* were found in approximately 30% of H, 33.33% of IBS and 20% of CD subjects, whereas 47.82% of UC and 66.67% of CRC patients fall into this category. Similar trends were observed for *F. prausnitzii* phylogroup II and *A. muciniphila* co-occurence analysis (*P* = 0.105), whereas percentages of each category were more similar between groups of subjects when analyzed in conjunction prevalence of total *F. prausnitzii* and *A. muciniphila* (*P* = 0.387). Of note, all subjects with IBS or CRC had at least one of the two species.

When analysing the cohort by IBD disease location, no significant differences within UC and within CD subtypes were achieved (Figure S1). However, a trend toward different proportions was observed. Half of subjects with distal UC (E2) were characterized by presenting only *A. muciniphila*. All patients with UC and also those with C-CD carried at least one species, whereas lower prevalences were found in CD patients with ileal involvement. Particularly, none of the two species were detected in 10–31% of CD patients with ileal disease location, whereas in 23.1% of I-CD and 40% of IC-CD, both species co-occurred. As the frequencies observed in C-CD patients resembled in some cases those in UC, we analyzed IBD subjects by grouping those with (either I-CD or IC-CD) or without (i.e., C-CD or UC) ileum involved (Figure 1B). Interestingly, when analyzing co-occurrence of *F. prausnitzii* phylogroup I and *A. muciniphila* the percentage of subjects with none or only one species detected was over 80% in those with ileal disease whereas in approximately 40% of subjects with colonic disease both species were found (*P* = 0.028).

When analyzing prevalence taking into account clinical data of the patients, no significant differences were observed by activity, medication or intestinal resection neither in CD nor in UC. No differences were found within CD patients according to disease behavior. Interestingly, *A. muciniphila* was not detected in any of the CD patients diagnosed with disease onset below 16 years of age (Figure 1C). It remains to be stablished if this is a common issue with UC patients, as none with early disease onset was included in our cohort.

### Abundances of mucosa-associated *A. muciniphila* and *F. prausnitzii*

*A. muciniphila*, total *F. prausnitzii* and its phylogroups load was compared amongst patients with different intestinal conditions (Table 3). Total *F. prausnitzii* was less abundant in CRC and IBD patients as compared to H subjects. However, statistically significant differences were achieved only for CRC (*P* = 0.028) and CD patients [*P* = 0.021, but not sustained after FDR assessment (Table S2)], probably because the reduction is lower for UC patients and the high variability between subjects. In CD patients, those with ileal involvement presented the lowest levels of this bacterium (*P* = 0.050), whereas IC-CD patients and C-CD were similar to UC (Table 3). Slight differences in average load were also found within UC patients although these differences were not statistically supported. Patients with ulcerative proctitis (E1) and extensive UC (E3) presented *F. prausnitzii* loads similar to H subjects, whereas those with E2 had abundances between CD patients and H subjects.

**Table 3:** Abundances of mucosa-associated *F. prausnitzii*, its phylogroups and *A. muciniphila* in controls (H), irritable bowel syndrome (IBS), colorectal cancer (CRC), Ulcerative Colitis (UC), and Crohn's disease (CD) patients.

	***n* patients**	F. prausnitzii^*^^§^	Phylogroup I^*^^§^	Phylogroup II^*^^§^	***A. muciniphila***
**H**	**17**	**5.07** ±**0.70**^a^	**3.00** ±**0.81**^a^	**2.32** ±**1.69**^ab^	**3.49** ±**0.81**
**IBS**	**3**	**5.50** ±**0.16**^ab^	**2.16** ±**1.54**^ab^	**2.00** ±**1.33**^ab^	**3.63** ±**0.95**
**CRC**	**3**	**4.24** ±**0.34**^b^	**1.12** ±**0.91**^bc^	**2.06** ±**1.62**^ab^	**2.84** ±**1.35**
**UC**	**23**	**4.82** ±**0.67**^ab^	**2.16** ±**1.26**^ac^	**2.79** ±**1.07**^a^	**3.07** ±**1.14**
**UC location**					
Ulcerative proctitis (E1)	5	5.01 ± 0.20	3.07 ± 0.32	3.44 ± 0.51	2.77 ± 1.36
Distal UC (E2)	11	4.77 ± 0.61	2.41 ± 1.11	2.74 ± 0.99	3.13 ± 1.24
Extensive UC or ulcerative pancolitis (E3)	6	5.03 ± 0.76	1.18 ± 1.36	2.69 ± 1.23	3.11 ± 0.98
**CD**	**31**	**4.22** ±**1.31**^b^	**1.13** ±**1.64**^b^	**1.47** ±**1.37**^b^	**3.19** ±**1.35**
**CD location**					
Ileal-CD (L1)	13	3.52 ± 1.33^•^	0.17 ± 1.10^•^	1.18 ± 1.55	2.87 ± 1.28
Colonic-CD (L2)	7	5.00 ± 0.93^■^	1.73 ± 1.94^■^	2.12 ± 1.36	3.33 ± 1.23
Ileocolonic-CD (L3)	10	4.47 ± 1.18^•■^	1.87 ± 1.60^■^	1.28 ± 1.09	3.42 ± 1.61
*p*-value group of subjects		**0.024**	**0.002**	**0.015**	0.540
*p*-value UC location		0.568	0.110	0.432	0.961
*p*-value CD location		**0.050**	**0.025**	0.349	0.538

§ Mean log_10_ (16S rRNA gene copies/million bacterial 16S rRNA gene copies) ± standard deviations.

* *Statistics was calculated separately for each variable (column). Only for those analyses statistically significant (P-value in bold), pairwise comparisons were conducted, and groups of patients with similar abundances are indicated with the same superscript (a, b). Disease locations of UC and CD patients have been analyzed as independent groups. Similarly, patients' subtypes with similar abundances are indicated with the same superscript (•■). In both cases, groups not sharing superscript are those with statistically different median abundance values (P-value < 0.05)*.

*F. prausnitzii* phylogroup I load was reduced in all groups of patients in comparison to H subjects. This reduction was particularly noticeable in CD and CRC patients (*P* < 0.008), while in UC and IBS patients, it was observed as well, but less apparent. When analyzing data by disease location, I-CD patients showed the most marked reduction of phylogroup I counts in comparison to other CD locations (*P* = 0.025). Values did not differ significantly in UC patients when analyzed by location. However, loads in E2 and E3 subjects resembled that of CD patients, while for E1 subjects, their profiles were closer to that observed in H subjects.

With respect to *F. prausnitzii* phylogroup II, its abundance was significantly reduced in CD patients when compared to UC subjects (*P* = 0.015) while similar loads were observed between all the other groups of subjects (Table 3). Although no differences by IBD location were found, loads tend to be lower in those with ileal involvement (either I-CD or IC-CD, *P* = 0.069).

**Figure 2:**
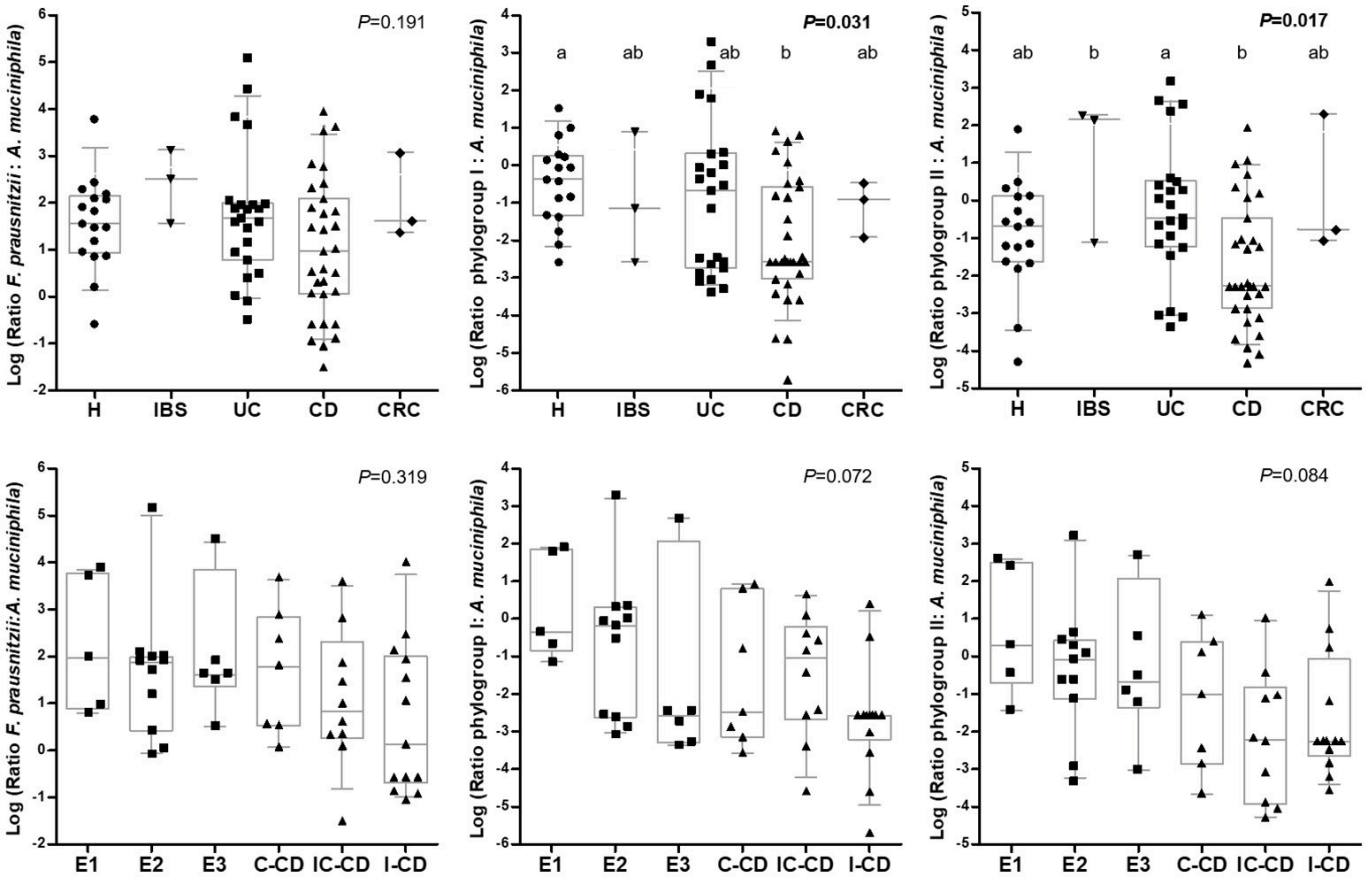
Box and whiskers plot of the ratio total *F. prausnitzii*: *A. muciniphila, F. prausnitzii* phylogroup I: *A. muciniphila*, and *F. prausnitzii* phylogroup II: *A. muciniphila:* (from left to right) by group of subjects (up) and by Inflammatory Bowel Disease subtype (down). Data are represented as log_10_ of each ratio. The median is represented by the horizontal line in each box. Boxes cover the 25 and 75% quantiles, and bars show the 10 and 90% quantiles. Individual data are also shown. Homogeneous subgroups in each plot are indicated with the same superscript. H, control subjects; IBS, irritable bowel syndrome; UC, ulcerative colitis; CD, Croh's disease; CRC, colorectal cancer; E1, ulcerative proctitis; E2, ulcerative left-sided colitis; E3, ulcerative pancolitis; IC-CD, ileocolonic CD, I-CD, ileal CD; C-CD, colonic CD. *A. muciniphila*, 16S rRNA gene *Akkermansia muciniphila; F. prausnitzii*, 16S rRNA gene total *Faecalibacterium prausnitzii;* Phylogroup I, 16S rRNA gene *F. prausnitzii* phylogroup I; Phylogroup II, 16S rRNA gene *F. prausnitzii* phylogroup II.

Interestingly, *A. muciniphila* load was similar between all the groups of subjects and also no differences were observed between IBD locations (*P* > 0.540; Table 3). In all groups of subjects, total *F. prausnitzii* counts outnumbered *A. muciniphila*, but there was a high variability between subjects, even within each condition. No difference in the ratio of total *F. prausnitzii*: *A. muciniphila* was found by group of subjects, neither when analyzing by IBD subtypes according to disease location. In contrast, when calculating these ratios by *F. prausnitzii* phylogroups, significant differences were found between conditions (Figure 2). CD patients, featured lower phylogroup I:*A. muciniphila* ratios than H (*P* = 0.031), and also lower phylogroup II: *A. muciniphila* ratios compared to UC (*P* = 0.017). When analyzing IBD groups by disease location, no significant differences were observed, probably due to the high dispersion of data and to the fact that when separating by location the number of patients included within each category is reduced. Nonetheless, subjects with a larger disease extension, or with ileum involvement, tended to feature lower values of both ratios. These differences in ratios are due to differences in *F. prausnitzii* (or phylogroup) load, as *A. muciniphila* load was similar across all subjects.

### *A. muciniphila* and *F. prausnitzii* abundances in relation to patients clinical and treatment data

No differences in *A. muciniphila*, counts were observed in either UC or CD patients according to activity status. Nevertheless, those patients with active UC featured the lowest load. *F. prausnitzii* and the abundance of the phylogroups did not differ between active and inactive UC patients (Table 4). CD patients with active disease feature lower levels of total *F. prausnitzii* and phylogroups in comparison to patients in remission, but differences did not achieve statistical significance either.

**Table 4:** *F. prausnitzii*, its phylogroups and *A. muciniphila* abundance in inflammatory bowel disease patients by disease activity status.

**Diagnostics^§^**	***n***	F. prausnitzii^*^	***P*-value**	Phylogroup I^*^	***P*-value**	Phylogroup II^*^	***P*-value**	A. muciniphila^*^	***P*-value**
**UC**			
Active	19	4.85 ± 0.71	0.464	2.12 ± 1.35	0.787	2.76 ± 1.11	0.523	3.04 ± 1.06	0.651
Inactive	3	4.61 ± 0.52		2.39 ± 0.97		3.18 ± 1.04		2.56 ± 1.29	
**CD**			
Active	18	4.10 ± 1.42	0.650	0.86 ± 1.45	0.373	1.36 ± 1.54	0.514	3.06 ± 1.28	0.514
Inactive	13	4.39 ± 1.19		1.52 ± 1.87		1.63 ± 1.15		3.36 ± 1.48	

Active CD and UC were defined when CDAI >150 (Best et al., 1976) and a Mayo score >3 (Pineton de Chambrun et al., 2010), respectively.

* Median (log_10_ 16S rRNA gene copies/million bacterial 16S rRNA gene copies) ± standard deviations.

§ *UC, ulcerative colitis; CD, Crohn's disease*.

Resection in CD patients was not a determining factor for *A. muciniphila* loads, either (Table 5). Instead, *F. prausnitzii* abundance was lower in those CD patients that underwent intestinal resection, with significant statistical differences for phylogroup II. Precisely, resected subjects had 10 times less phylogroup II than those without intestinal surgery (*P* = 0.018) whereas the phylogroup I load was only slightly lower in resected than non-resected patients.

**Table 5:** *F. prausnitzii*, its phylogroups and *A. muciniphila* abundance in inflammatory bowel disease patients depending on whether or not they have had intestinal resection during the course of the disease.

**Diagnostics^§^**	***n***	F. prausnitzii*	***P*-value**	Phylogroup I*	***P*-value**	Phylogroup II*	***P*-value**	A. muciniphila*	***P*-value**
**UC**			
Non-resected	19	4.73 ± 0.68	na	1.97 ± 1.21	na	2.79 ± 1.01	0.544	3.05 ± 1.14	na
Resected	1	4.91		3.45		2.68		4.11	
**CD**			
Non-resected	21	4.46 ± 1.33	0.239	1.33 ± 1.84	0.842	1.85 ± 1.40	**0.018**	3.29 ± 1.44	0.476
Resected	6	3.89 ± 1.04		1.07 ± 1.33		0.59 ± 0.44		3.46 ± 0.45	

* Median (log_10_ 16S rRNA gene copies/million bacterial 16S rRNA gene copies) ± standard deviations; na, not applicable. Analyses statistically significant are highlighted in boldface.

§ *UC, ulcerative colitis; CD, Crohn's disease*.

The *A. muciniphila* load was lower in CD patients who presented with the disease below 16 years of age (Table 6). This group of patients also featured very low quantities of *F. prausnitzii* phylogroup I although statistical significance was not achieved. No differences in these bacterial loads were observed between groups of UC patients with different age of disease onset. We also analyzed disease duration, but no statistically significant correlation was found between any of the bacterial loads and time of disease duration (data not shown).

**Table 6:** *F. prausnitzii*, its phylogroups and *A. muciniphila* abundances in inflammatory bowel disease patients by age of disease onset.

**Diagnostics^§^**	***n***	F. prausnitzii^*^	***P*-value**	Phylogroup I^*^	***P*-value**	Phylogroup II^*^	***P*-value**	A. muciniphila^*^^#^	***P*-value**
**UC**			
17–40 years (A2)	11	4.87 ± 0.47	0.676	2.52 ± 1.09	0.171	3.12 ± 0.88	0.305	2.93 ± 1.19	0.569
>41 years (A3)	9	4.64 ± 0.91		1.65 ± 1.42		2.58 ± 1.10		2.88 ± 1.07	
**CD**			
<16 years (A1)	5	3.62 ± 1.59		0.004 ± 0.44		1.65 ± 1.71		1.76 ± 0.73^a^	
17–40 years (A2)	21	4.26 ± 1.36	0.562	1.40 ± 1.87	0.112	1.30 ± 1.34	0.547	3.31 ± 1.16^b^	**0.030**
>41 years (A3)	3	4.67 ± 0.71		0.97 ± 0.48		2.26 ± 1.47		4.20 ± 2.15^b^	

* Median (log_10_ 16S rRNA gene copies/million bacterial 16S rRNA gene copies) ± standard deviations.

§ UC, ulcerative colitis; CD, Crohn's disease.

# *Statistics was calculated separately for each variable (column). Groups of patients with similar abundances of A. muciniphila are indicated with the same superscript (a, b) whereas groups not sharing superscript are those with statistically different median abundance values (P < 0.05). Analyses statistically significant are highlighted in boldface*.

Finally, data were analyzed by taking into account the medication of the patients at the time of sampling (Table 7). No differences in *A. muciniphila, F. prausnitzii* or in phylogroups abundances were observed between medications for any IBD. However, those UC patients that received anti-tumor necrosis factor had the lowest levels of *A. muciniphila*. In contrast, those CD patients receiving moderate immunosupressants had lower *F. prausnitzii* loads than patients without treatment or receiving therapies such as mesalazine or anti-tumor necrosis factor.

**Table 7:** *F. prausnitzii*, its phylogroups and *A. muciniphila* abundances in inflammatory bowel disease by medication at sampling.

**Diagnostics^§^**	***n***	F. prausnitzii^*^	***P*-value**	Phylogroup I^*^	***P*-value**	Phylogroup II^*^	***P*-value**	***A. muciniphila***	***P*-value**
**UC**			
No treatment	11	4.81 ± 0.72		1.94 ± 1.35		2.74 ± 1.13		3.10 ± 1.11	
Mesalazine	3	4.87 ± 0.40	0.783	2.30 ± 0.97	0.578	3.20 ± 1.01	0.639	3.53 ± 1.82	0.387
Mod. Immsup	4	4.95 ± 0.61		2.97 ± 0.33		3.18 ± 1.54		3.06 ± 0.51	
Anti-TNF	3	4.35 ± 0.96		1.53 ± 1.85		2.37 ± 1.03		2.10 ± 1.52	
**CD**			
No treatment	10	4.30 ± 1.51		1.04 ± 1.96		1.67 ± 1.61		2.69 ± 1.08	
Mesalazine	3	5.00 ± 0.41	0.537	1.30 ± 1.67	0.975	2.24 ± 1.89	0.719	3.90 ± 2.20	0.125
Mod. Immsup	10	3.84 ± 1.21		0.99 ± 1.50		1.15 ± 1.09		3.72 ± 0.97	
Anti-TNF	6	4.31 ± 1.68		1.42 ± 1.76		1.60 ± 1.42		2.72 ± 1.78	

* Median (log_10_ 16S rRNA gene copies/million bacterial 16S rRNA gene copies) ± standard deviations.

§ *UC, ulcerative colitis; CD, Crohn's disease; Mod. Immsup, moderate immunosuppresants; Anti-TNF, Anti-tumor necrosis factor*.

### Correlation between *A. muciniphila* and *F. prausnitzii* abundances

**Figure 3:**
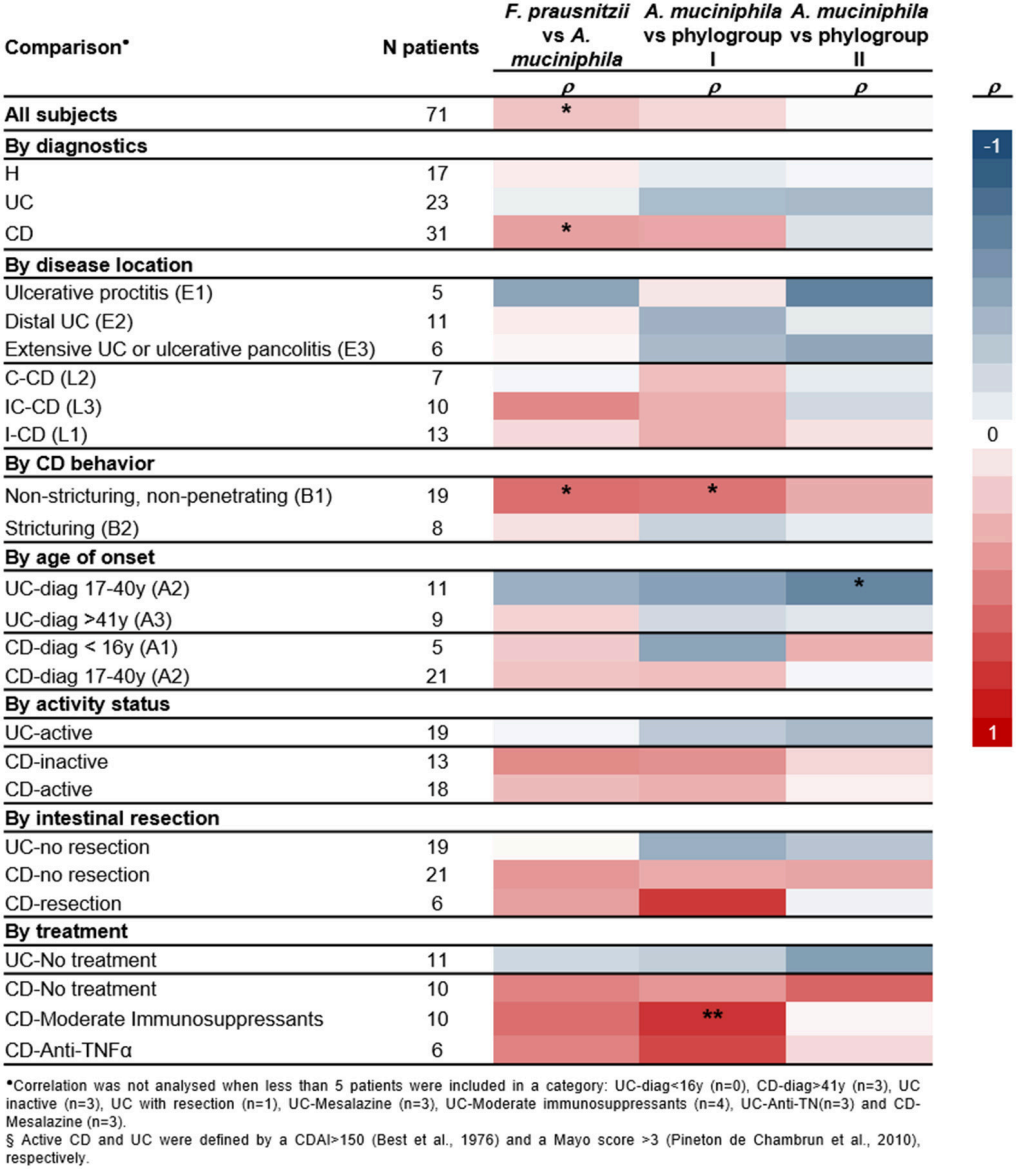
Heatmap of spearman correlation cofficients between *A. muciniphila*, total *F. prausnitzii* and its phylogroups abundances, splitting up patients by diagnostics, inflammatory bowel disease subtypes, and main clinical characteristics. Significant correlations are indicated ^*^P < 0.05, ^**^P < 0.01.

Correlation between *A. muciniphila* and *F. prausnitzii* numbers was analyzed to provide supporting evidence for a direct/indirect effect of one population over the other or about a putative common factor affecting both species populations in a given condition (Figure 3).

No correlation between these two species was found in H or UC patients (Figure S3). Therefore, in these conditions, factors of the gut environment may be differentially impacting on each species. In contrast, positive correlation of both species load was observed in CD subjects (Figure 3). Analysis by phylogroups indicated that *A. muciniphila* quantity tended to positively correlate with phylogroup I in CD patients (*P* = 0.060), whereas no significant correlation was observed with phylogroup II (Figure S3).

Moreover, no significant correlation between the two species was observed, when splitting subjects by activity status or whether or not they have had intestinal resection. Of note, a positive correlation between *A. muciniphila* and *F. prausnitzii* (particularly phylogroup I) was observed in CD patients with non-stricturing, non-penetrating disease (B1) and in those under moderate immunosuppressants (ρ ≥ 0.539, *P* ≤ 0.017). In contrast, in UC patients with disease onset between 17 and 40 years of age, a negative correlation between *A. muciniphila* and *F. prausnitzii* phylogroup II was observed (ρ = −0.673, *P* = 0.023).

## Discussion

*A. muciniphila* and *F. prausnitzii* are two symbiotic and numerically abundant members of the gut microbiota, and both have been associated with dysbiosis in several disease conditions, including IBD. Their niche is close to the intestinal mucosa, and therefore it can be hypothesized that they may play a key role in cross-talk with the host. Both species are considered to have a part in a well-functioning gut and thus are considered as promising next generation probiotics (Neef and Sanz, 2013; Martín et al., 2017). In the present study we have analyzed the prevalence and abundance of mucosa associated *A. muciniphila*, total *F. prausnitzii* and phylogroup in H and IBD subjects, taking into account the diversity of disease locations and the clinical features of patients. Some IBS and CRC have been included as well, but only for illustrative purposes given the low number of patients engaged. The abundance of both species has been previously reported to be reduced in several intestinal disorders (Belzer and de Vos, 2012; Miquel et al., 2013), but here for the first time we correlate the load of both species. Through analysis of clinical data, we consider which particular conditions this underrepresentation is favored, and whether or not the imbalance of one species is linked to changes in the abundance of the other.

Our data show that the *A. muciniphila* load in the mucosa of H subjects is slightly higher (2.0- to 4.5-fold, respectively) than in IBD and CRC patients, but is not statistically significant. An increase in *A. muciniphila* abundance in CRC patients compared to controls has been previously found in stools (Weir et al., 2013) but not in mucosal biopsies (Mira-Pascual et al., 2015), and the analysis of our limited cohort is in line with this finding. Previous studies have reported a significant decrease of this species in IBD subjects (Png et al., 2010). Methodological differences may explain the inconsistency with our findings, as we exclusively focused on colonic samples. In biopsy samples, Png and collaborators observed a reduction of this species in IBD patients that ranged between 2.9- and 3.9-fold when compared to controls, which is similar to the reduction observed in our subjects. In that study (Png et al., 2010), differences were observed depending on whether or not the tissue was affected, with the depletion being more conspicuous in inflamed tissue, and without reaching significant differences between non-inflamed tissue of CD and controls, which is in line with our results as we used non-affected tissue. In addition, we have explored differences taking into account disease location, activity or intestinal resection, but no association between *A. muciniphila* load and these variables has been revealed. Intriguingly, CD patients who presented with disease below 16 years of age had a striking reduction of this species compared to those with disease onset at a later age. *A. muciniphila* has been reported to colonize the gut in early infancy, and loads in infants 1 year old are similar to that found in adults (Collado et al., 2007). Therefore, it seems likely that this depletion is not a general phenomenon that occurs in IBD or age-driven, but due to particular features of pediatric IBD that are sustained throughout the disease. In line with this, discrepancies between dysbiosis signatures in adult and infant IBD patients have been previously reported (Hansen et al., 2012) and it remains to be explored through prospective studies if early disease onset results in long term microbial signatures. Another future application of this finding could be to explore the usefulness of *A. muciniphila* depletion as a biomarker to assist in pediatric IBD diagnosis.

Regarding mucosa-associated *F. prausnitzii* loads we have observed a marked reduction in CRC and CD patients, especially in those with ileal involvement, affecting both phylogroups of this species. Although less prominent, UC patients also featured lower *F. prausnitzii* abundance than H subjects. Our study is in agreement with previous reports which found *F. prausnitzii* to be reduced in CRC and IBD adults (Swidsinski et al., 2005, 2008; Martinez-Medina et al., 2006; Frank et al., 2007; Sokol et al., 2008, 2009; Willing et al., 2009; McLaughlin et al., 2010; Vermeiren et al., 2012; Kabeerdoss et al., 2013; Machiels et al., 2013; Miquel et al., 2013; Lopez-Siles et al., 2014, 2016). Besides, lower abundance of both *F. prausnitzii* phylogroups has been previously reported concerning CD patients (Jia et al., 2010; Lopez-Siles et al., 2016), which is in line with our findings. Moreover, because we have observed differences between IBD subtypes, our results support the hypothesis that patients with ileal disease location constitute a differentiated pathological entity (Willing et al., 2009). We have corroborated that the reduction of *F. prausnitzii* numbers compared to H subjects takes place in both active and inactive IBD patients (Willing et al., 2009), with active CD patients featuring the lowest levels of phylogroup I. Also in agreement with previous studies (Sokol et al., 2008) lower numbers of *F. prausnitzii* were detected in resected CD patients, but in our study, statistically significant differences were only achieved for phylogroup II, probably because the depletion was more striking. It remains unknown why there are shifts in particular subgroups of this species. To date, several articles convey the point that the genus *Faecalibacterium* hosts a complex diversity (Lopez-Siles et al., 2015; Benevides et al., 2017; Martín et al., 2017). This diversity has been shown mainly through phylogenetic methods, but phenotypical diversity also exists. Supporting this point, studies characterizing several strains of this species isolated from different origins have failed to find phenotypic traits that consistently distinguish members from one or other subtype (Lopez-Siles et al., 2012; Foditsch et al., 2014; Martín et al., 2017). However, the effect of host factors differentially influencing *F. prausnitzii* subpopulations has been poorly explored which may explain our results. Another hypothesis could be that subtypes of *F. prausnitzii* interact in a different manner with other members of the microbiome, which has also been scarcely studied to date.

We have explored co-occurrence and correlation between *A. muciniphila* and *F. prausnitzii* in H and IBD patients. We considered that both species may have a syntrophic relationship, thus we hypothesize that the depletion or enrichment of one would imply the same effect on the other. In particular, *A. muciniphila* mucolytic activity could release oligosaccharides, co-factors, vitamins, and short chain fatty acids, including acetate that juxtaposed species could use for growth. Indeed, *F. prausnitzii* has been proven to be able to use some oligosaccharides derived from mucus and its growth is stimulated by acetate and requires presence of vitamins in the medium (Duncan et al., 2002; Lopez-Siles et al., 2012). These compounds, can be provided by *A. muciniphila*, although not exclusively, and therefore establish cross-feeding interactions. A recent study based on co-culture experiments demonstrated this trophic interaction (Belzer et al., 2017). However, in most of the cases studied here, we did not find a correlation between *F. prausnitzii* and *A. muciniphila* abundances, and the two species co-occurred only in 41.5% of subjects engaged in the study. This may be because *F. prausnitzii* does not depend exclusively on by-products synthesized by *A. muciniphila*. In agreement with that, other studies have reported that *F. prausnitzii* can benefit from the presence of a variety of acetate-producing species (Wrzosek et al., 2013; Rios-Covian et al., 2015). It would be interesting to determine whether this species (or other mucus-inhabiting species) increase in patients in which *A. muciniphila* diminishes, and thus may be partially replacing its role concerning acetate production.

Nonetheless, we observed that positive correlation between the two species happens in CD patients, and particularly for those with B1 behavior or under immunosuppressant therapy. The fact that there is a positive correlation of the two species indicates that their abundance varies in a similar way in this particular condition. The most likely scenario is that in CD the two bacteria are similarly affected by host and gut environmental factors. To support this hypothesis, both species share the characteristic that their growth is severely compromised at pH < 5.5 (Derrien et al., 2004; Lopez-Siles et al., 2012) and in turn, acidic stools have been reported for IBD patients (Nugent et al., 2001; Barkas et al., 2013). Notably, in those cases of correlation between both species, only members of phylogroup I were involved. It would be interesting to perform co-culture studies with different *F. prausnitzii* strains, and monitor their growth under different conditions in order to determine more accurately their relationship.

In our cohort, *A. muciniphila* was not detected in 57.1% of all subjects but this seems to be related to the fact that this species has a lower relative abundance in the gut, rather than to a higher sensitivity to gut disease. In contrast, *A. muciniphila* was more frequently found in UC patients. This could be partially explained by the fact that a higher proportion of loose mucus has been found in UC patients (Antoni et al., 2014), which is the likely niche for *A. muciniphila*. Another hypothesis that can not be ruled out is that some factor of UC patients favors the presence of *A. muciniphila*. In our limited cohort, we have also observed higher prevalence of this species in CRC group compared to controls, which is in line with previous findings (Mira-Pascual et al., 2015). However, our data points out that this higher presence does not imply an increase in the abundance at the mucosal level. In contrast, almost 90% of subjects were *F. prausnitzii* carriers and thus the fact that it is a second-liner in the mucosa and the fact that this species can rely on other members of the gut microbiota for cross-feeding may explain its higher ubiquity and abundance compared to *A. muciniphila*.

Finally, our study revealed that CD patients are characterized by a low *F. prausnitzii*: *A. muciniphila*, ratio affecting both phylogroups. This indicates that compared to H and UC, these patients have an altered proportion of beneficial microorganisms in the mucosa. Although our study does not allow us to decipher if this imbalance is a cause or a consequence of the disease, it can be an aggravator because the two species have been linked to be key for mucus integrity (Derrien et al., 2004; Wrzosek et al., 2013) and gut homeostasis. A significant depletion of both species has also been reported in children with atopic disease (Candela et al., 2012), and therapeutic strategies to restore these species needs to be explored, particularly for disorders that have in common to feature chronic inflammation. In addition, two recent studies have linked the two species with response to immunotherapy treatment (Gopalakrishnan et al., 2017; Routy et al., 2017), thus pointing out another situation in which it is relevant to have these bacteria. Further studies to assess implications in IBD treatment response would be interesting, as immunomodulators are among the usual therapies prescribed to IBD patients. Finally, further confirmation of our results in a larger cohort would be required given that we have engaged a limited number of subjects, and thus it would provide robustness to those findings not sustained after FRD assessment.

## Conclusions

IBD patients are characterized by a reduction of *F. prausnitzii* and a slight underrepresentation of *A. muciniphila* in the colonic mucosa, regardless of disease activity status. While differences in *F. prausnitzii* load have been observed for I-CD patients, early onset CD is characterized by a lack of *A. muciniphila*, but further prospective studies are required to assess if this feature is sustained long term. Positive correlation between the two species was found in CD patients, and further studies are required to elucidate which common factors alter both populations in particular gut disorders.

## Author contributions

XA, SD, LG-G, ML-S, and MM-M study concept and design. XA, NE-C, ML-S, and MS-M acquisition of data. ML-S and MM-M interpretation of data and statistical analysis. ML-S drafting the manuscript. XA, SD, NE-C, LG-G, MM-M, and MS-M critical revision of the manuscript for important intellectual content. LG-G and MM-M obtained funding. All authors have approved the final version of the manuscript and agree to be accountable for all aspects of the work, ensuring that questions related to the accuracy or integrity of any part of the work are appropriately investigated and resolved.

### Conflict of interest statement

XA is a consultant for AbbVie, Janssen and Takeda, and has received honoraria for lectures, including services on speakers bureaus from AbbVie, MS-D, Janssen, Takeda, Shire, Zambon and Ferring. XA, LG-G, ML-S, and MM-M, have filed a European patent for a “Method for the detection, follow up and/or classification of intestinal diseases” (application number EP15382427). The remaining authors declare that the research was conducted in the absence of any commercial or financial relationships that could be construed as a potential conflict of interest.
