# Supplementary material for: Alterations in the Abundance and Co-occurrence of Akkermansia muciniphila and Faecalibacterium prausnitzii in the Colonic Mucosa of Inflammatory Bowel Disease Subjects
Source: Front Cell Infect Microbiol. 2018 Sep 7;8:281. doi: 10.3389/fcimb.2018.00281 (PMC6137959; doi:10.3389/fcimb.2018.00281)
Supplement: Supplementary file 1 [file Table_1.DOCX]

Supplementary Material

**Alterations in the abundance and co-occurrence of *Akkermansia muciniphila* and *Faecalibacterium prausnitzii* in the colonic mucosa of inflammatory bowel disease subjects**

Mireia Lopez-Siles, Núria Enrich-Capó, Xavier Aldeguer, Miriam Sabat-Mir, Sylvia H. Duncan, L. Jesús Garcia-Gil^1^, Margarita Martinez-Medina

*** Correspondence:** L. Jesús Garcia-Gil, [jesus.garcia@udg.edu](mailto:jesus.garcia@udg.edu)

# Supplementary Tables

**Table S1.** Mean 16S rRNA gene copies ± standard deviations for *F. prausnitzii*, its phylogroups, *A. muciniphila* and total bacteria quantifications, found per μl of DNA extract.

| **Id Biopsy** | ***F. prausnitzii*** | | | **Phylogroup I** | | | **Phylogroup II** | | | ***A. muciniphila*** | | | **Total bacteria** | | |
| --- | --- | --- | --- | --- | --- | --- | --- | --- | --- | --- | --- | --- | --- | --- | --- |
|  | **n replicates** | **mean** | **SD** | **n replicates** | **mean** | **SD** | **n replicates** | **mean** | **SD** | **n replicates** | **mean** | **SD** | **n replicates** | **mean** | **SD** |
| 6 | 3 | 3.94E+05 | 9.99E+03 | 3 | 1.42E+01 | 3.15E-01 | 3 | 3.88E+03 | 5.62E+01 | 3 | 1.49E+02 | 7.06E+00 | 2 | 3.49E+06 | 3.03E+05 |
| 19 | 3 | 4.83E+04 | 2.80E+03 | 2 | undet | - | 2 | undet | - | 2 | 1.36E+05 | 6.50E+02 | 2 | 1.77E+06 | 1.18E+05 |
| 77 | 3 | 1.13E+03 | 1.47E+01 | 3 | undet | - | 3 | 2.44E+00 | 8.16E-02 | 3 | 3.23E+04 | 7.75E+02 | 2 | 2.67E+06 | 2.61E+05 |
| 96 | 3 | 1.23E+04 | 8.97E+02 | 3 | undet | - | 3 | undet | - | 3 | undet | - | 2 | 3.45E+05 | 3.03E+04 |
| 99 | 3 | 1.29E+06 | 4.45E+04 | 2 | 1.65E+03 | 1.11E+01 | 2 | 3.67E+03 | 1.58E+01 | 3 | undet | - | 2 | 3.50E+06 | 5.44E+04 |
| 115 | 3 | undet | - | 3 | undet | - | 3 | undet | - | 3 | undet | - | 3 | 2.71E+06 | 1.39E+05 |
| 195 | 3 | 1.21E+05 | 1.05E+04 | 3 | undet | - | 3 | undet | - | 3 | undet | - | 3 | 4.15E+05 | 5.43E+03 |
| 216 | 3 | 2.75E+03 | 4.49E+01 | 3 | undet | - | 3 | 9.30E+00 | 3.03E-01 | 3 | undet | - | 3 | 5.06E+04 | 6.41E+02 |
| 228 | 3 | 2.40E+05 | 1.69E+04 | 3 | 3.33E+02 | 7.14E+00 | 3 | 4.23E+03 | 5.55E+01 | 3 | 2.65E+04 | 8.83E+02 | 2 | 1.20E+06 | 3.60E+04 |
| 232 | 3 | 7.54E+03 | 3.49E+02 | 3 | 8.03E+01 | 2.04E+00 | 3 | 0.00E+00 | 9.01E-06 | 3 | 3.09E+02 | 6.01E+00 | 2 | 5.85E+04 | 2.90E+03 |
| 240 | 3 | 2.85E+03 | 2.77E+02 | 3 | 5.53E+01 | 5.25E-01 | 3 | 2.24E+01 | 3.00E-01 | 3 | undet | - | 2 | 4.62E+04 | 1.08E+03 |
| 243 | 3 | 2.55E+04 | 1.86E+03 | 3 | 2.40E+03 | 1.09E+01 | 3 | 4.90E+02 | 1.06E+01 | 3 | undet | - | 2 | 1.05E+05 | 1.04E+04 |
| 277 | 3 | 8.87E+04 | 2.99E+03 | 3 | 1.23E+03 | 1.28E+01 | 3 | 2.18E+03 | 2.14E+01 | 3 | 4.01E+03 | 1.70E+02 | 2 | 2.81E+05 | 1.85E+04 |
| 278 | 3 | 1.48E+03 | 9.64E+01 | 3 | undet | - | 3 | undet | - | 3 | undet | - | 2 | 3.03E+04 | 5.96E+02 |
| 304 | 3 | 1.12E+04 | 1.23E+02 | 3 | 1.81E+01 | 1.92E-01 | 3 | 1.55E+01 | 1.23E-01 | 2 | 4.65E+04 | 1.55E+02 | 2 | 1.16E+05 | 2.68E+02 |
| 309 | 3 | undet | - | 3 | undet | - | 3 | undet | - | 3 | undet | - | 2 | 7.51E+05 | 2.16E+04 |
| 427 | 3 | 4.62E+04 | 1.81E+03 | 3 | undet | - | 3 | 1.87E+03 | 0.00E+00 | 3 | undet | - | 2 | 2.07E+06 | 1.36E+04 |
| 431 | 3 | 3.45E+03 | 4.36E+01 | 3 | 7.02E+00 | 1.38E-01 | 3 | undet | - | 3 | 1.95E+01 | 4.88E-01 | 2 | 1.10E+05 | 6.83E+03 |
| 443 | 3 | 1.48E+02 | 9.04E+00 | 3 | undet | - | 3 | undet | - | 2 | 8.63E+03 | 3.98E+01 | 2 | 1.18E+05 | 9.52E+03 |
| 451 | 3 | undet | - | 3 | undet | - | 3 | undet | - | 3 | undet | - | 2 | 1.44E+05 | 8.60E+03 |
| 490 | 3 | 5.10E+04 | 4.20E+03 | 3 | 3.31E+03 | 4.46E+01 | 3 | undet | - | 2 | 7.96E+04 | 7.20E+02 | 3 | 4.19E+04 | 1.84E+03 |
| 498 | 3 | 8.00E+04 | 4.82E+03 | 3 | 2.38E+03 | 3.66E+00 | 3 | 4.59E+02 | 6.34E+00 | 3 | undet | - | 3 | 1.15E+05 | 2.23E+03 |
| 504 | 3 | 3.15E+04 | 4.84E+02 | 3 | 1.58E+03 | 4.62E+01 | 3 | 5.07E+02 | 2.42E+01 | 2 | 5.39E+02 | 2.82E+00 | 2 | 8.12E+04 | 5.60E+03 |
| 510 | 3 | undet | - | 3 | 2.97E+00 | 1.35E-01 | 3 | 2.00E-02 | 3.20E-04 | 3 | undet | - | 2 | 1.41E+04 | 2.39E+02 |
| 514 | 3 | 3.49E+03 | 2.14E+02 | 3 | 3.26E+02 | 1.13E+01 | 3 | 1.07E+02 | 3.60E+00 | 3 | undet | - | 2 | 4.89E+04 | 1.85E+03 |
| 521 | 3 | 1.10E+04 | 4.45E+02 | 3 | 9.82E+02 | 1.43E+01 | 3 | 4.47E+01 | 3.23E+00 | 3 | 2.37E+03 | 4.74E+02 | 3 | 4.85E+06 | 7.13E+04 |
| 526 | 3 | 3.62E+03 | 1.54E+02 | 3 | 2.20E+01 | 8.30E-01 | 3 | 2.84E+01 | 9.85E-01 | 3 | 4.44E+01 | 1.76E+00 | 2 | 1.25E+04 | 8.98E+02 |
| 530 | 3 | 9.53E+03 | 7.65E+02 | 3 | 1.14E+02 | 1.10E+00 | 3 | 3.25E+01 | 1.08E+00 | 3 | 4.68E+01 | 9.18E-01 | 3 | 7.53E+04 | 1.67E+03 |
| 539 | 3 | 4.53E+04 | 2.13E+03 | 3 | 5.10E+01 | 1.07E+00 | 3 | 1.03E+02 | 6.29E-01 | 2 | undet | - | 2 | 2.83E+04 | 2.06E+01 |
| 542 | 3 | 1.12E+04 | 1.91E+02 | 3 | 1.32E+00 | 1.47E-01 | 3 | 3.50E-01 | 1.51E-02 | 2 | undet | - | 3 | 8.56E+04 | 8.61E+02 |
| 547 | 3 | 4.07E+03 | 9.75E+01 | 3 | undet | - | 3 | undet | - | 2 | undet | - | 2 | 3.73E+05 | 8.72E+02 |
| 554 | 3 | 5.10E+03 | 3.03E+02 | 3 | 5.47E+01 | 7.31E-02 | 3 | 4.88E+01 | 9.89E-01 | 3 | 1.05E+04 | 3.44E+02 | 2 | 1.16E+04 | 3.59E+02 |
| 560 | 3 | 3.61E+04 | 7.87E+02 | 3 | 1.01E+02 | 2.06E+00 | 3 | 4.28E+01 | 2.66E+00 | 2 | 1.07E+05 | 3.58E+02 | 2 | 5.53E+04 | 1.99E+03 |
| 572 | 3 | 5.86E+04 | 1.99E+03 | 3 | 6.20E-01 | 5.49E-02 | 3 | 4.32E+02 | 9.96E+00 | 3 | 4.90E+03 | 5.23E+01 | 2 | 2.18E+05 | 1.98E+04 |
| 574 | 3 | 1.42E+05 | 3.32E+03 | 3 | undet | - | 3 | 2.23E+02 | 2.32E+00 | 2 | 6.38E+03 | 2.80E+01 | 2 | 3.43E+05 | 1.57E+04 |
| 577 | 3 | 1.51E+04 | 1.21E+03 | 3 | 5.26E+02 | 1.84E+01 | 3 | 8.95E+01 | 2.40E+00 | 3 | 8.32E+03 | 1.12E+02 | 2 | 1.87E+05 | 3.47E+03 |
| 580 | 3 | 2.08E+03 | 3.14E+01 | 3 | 1.56E+00 | 3.19E-02 | 3 | 8.60E-01 | 1.64E-02 | 3 | 6.44E+03 | 1.29E+02 | 2 | 1.38E+05 | 1.01E+04 |
| 594 | 3 | 5.93E+04 | 2.13E+03 | 3 | 7.39E+02 | 1.89E+01 | 3 | 5.20E+02 | 7.23E-01 | 3 | undet | - | 2 | 3.38E+05 | 2.30E+04 |
| 602 | 3 | 1.58E+04 | 1.17E+03 | 3 | 1.66E+02 | 1.44E+00 | 3 | 9.02E+01 | 1.42E+00 | 3 | 1.48E+03 | 4.13E+01 | 3 | 3.96E+05 | 2.92E+04 |
| 610 | 3 | 6.09E+05 | 1.63E+04 | 3 | 3.31E+03 | 1.06E+02 | 3 | 8.02E+03 | 4.92E+01 | 3 | 3.36E+02 | 8.02E+00 | 2 | 9.68E+05 | 3.22E+04 |
| 618 | 3 | 3.48E+03 | 3.02E+02 | 3 | 1.31E+00 | 1.07E-01 | 3 | undet | - | 3 | 9.29E+01 | 1.02E+00 | 2 | 1.05E+04 | 7.93E+02 |
| 624 | 3 | 1.44E+05 | 1.11E+04 | 3 | 2.00E+03 | 5.64E+01 | 3 | undet | - | 3 | 1.62E+04 | 1.74E+02 | 2 | 2.52E+06 | 1.75E+05 |
| 670 | 3 | 2.41E+05 | 1.11E+04 | 3 | 7.83E+02 | 1.36E+01 | 3 | 5.93E+02 | 5.15E+00 | 3 | 8.82E+03 | 7.07E+02 | 2 | 6.28E+05 | 1.98E+04 |
| 706 | 3 | undet | - | 3 | undet | - | 3 | undet | - | 3 | 1.25E+01 | 3.22E-01 | 3 | 5.17E+05 | 1.29E+04 |
| 731 | 3 | 4.85E+04 | 2.64E+03 | 3 | 2.15E+01 | 3.99E-01 | 3 | 8.44E+01 | 3.21E+00 | 2 | 5.40E+03 | 2.32E+01 | 3 | 1.69E+05 | 1.96E+03 |
| 752 | 3 | 9.92E+04 | 5.92E+03 | 3 | undet | - | 3 | 6.01E+02 | 2.82E+01 | 3 | undet | - | 2 | 1.35E+06 | 1.72E+04 |
| 762 | 3 | 2.65E+04 | 9.66E+02 | 3 | undet | - | 3 | 7.56E+01 | 1.73E+00 | 2 | 2.15E+01 | 4.46E-02 | 2 | 2.88E+04 | 2.92E+02 |
| 782 | 3 | 1.37E+04 | 1.50E+02 | 3 | 2.68E+01 | 5.43E-02 | 3 | 2.64E+00 | 1.05E-01 | 3 | undet | - | 2 | 2.88E+04 | 2.36E+03 |
| 790 | 3 | 4.71E+05 | 1.33E+04 | 3 | 7.64E+03 | 8.42E+01 | 3 | 5.94E+03 | 4.60E+01 | 3 | 1.31E+01 | 7.40E-01 | 2 | 1.69E+06 | 2.08E+04 |
| 809 | 3 | 3.65E+04 | 2.58E+03 | 3 | 6.29E+02 | 7.71E+00 | 3 | 6.22E+02 | 1.38E+01 | 3 | 4.56E+00 | 6.13E-01 | 2 | 8.34E+04 | 1.98E+03 |
| 813 | 3 | 5.31E+05 | 2.56E+04 | 3 | 3.08E+03 | 5.60E+01 | 3 | 5.03E+03 | 1.60E+02 | 3 | 1.35E+03 | 1.51E+02 | 3 | 2.34E+06 | 4.59E+04 |
| 833 | 3 | 4.90E+03 | 3.33E+02 | 3 | 8.79E+00 | 2.12E-01 | 3 | 1.54E+01 | 4.22E-01 | 2 | 8.04E+03 | 2.43E+01 | 3 | 2.98E+04 | 4.06E+03 |
| 837 | 3 | 1.08E+03 | 4.50E+01 | 3 | undet | - | 3 | 9.65E+01 | 2.39E-01 | 3 | 1.42E+03 | 4.78E+02 | 3 | 4.11E+04 | 1.93E+03 |
| 841 | 3 | 5.27E+04 | 4.00E+03 | 3 | 3.55E+02 | 5.05E+00 | 3 | 2.25E+03 | 2.87E+01 | 3 | 1.84E+03 | 8.12E+02 | 3 | 2.33E+05 | 1.77E+04 |
| 916 | 3 | 1.23E+03 | 8.61E+01 | 3 | 2.60E-01 | 1.23E-02 | 3 | 8.00E-02 | 7.61E-03 | 3 | undet | - | 3 | 4.93E+04 | 5.71E+02 |
| 921 | 3 | 2.22E+04 | 2.26E+02 | 3 | undet | - | 3 | 1.82E+03 | 3.11E+01 | 3 | 1.84E+03 | 6.33E+02 | 3 | 4.29E+05 | 7.99E+03 |
| 925 | 3 | 1.87E+04 | 1.08E+03 | 3 | 8.75E+02 | 1.17E+01 | 3 | 4.53E+02 | 1.71E+01 | 3 | 1.33E+03 | 3.23E+02 | 2 | 6.81E+05 | 5.31E+04 |
| 930 | 3 | 1.16E+05 | 1.37E+03 | 3 | 5.68E+02 | 8.61E+00 | 3 | 2.48E+03 | 1.80E+01 | 3 | 4.32E+03 | 1.46E+02 | 2 | 6.63E+05 | 9.05E+03 |
| 934 | 3 | 7.07E+02 | 6.21E+01 | 3 | undet | - | 3 | undet | - | 3 | 3.55E+01 | 2.83E+00 | 3 | 7.99E+04 | 2.44E+03 |
| 940 | 3 | 3.09E+04 | 1.20E+03 | 3 | 3.62E+02 | 2.11E-01 | 3 | 1.11E+03 | 2.61E+01 | 3 | 1.54E+01 | 8.03E-01 | 2 | 3.50E+05 | 2.29E+04 |
| 945 | 3 | 1.27E+02 | 4.88E+00 | 3 | undet | - | 3 | undet | - | 3 | undet | - | 3 | 7.38E+04 | 9.50E+02 |
| 950 | 3 | 1.51E+04 | 1.19E+03 | 3 | 1.98E+02 | 2.84E+00 | 3 | 1.23E+03 | 2.96E+01 | 3 | 1.10E+01 | 1.49E+00 | 3 | 9.46E+04 | 1.20E+03 |
| 956 | 3 | 3.41E+03 | 1.34E+02 | 2 | 2.67E+01 | 2.12E-01 | 2 | 1.31E+02 | 2.47E-01 | 3 | undet | - | 2 | 5.99E+04 | 1.83E+03 |
| 960 | 3 | 5.09E+01 | 2.40E+00 | 3 | undet | - | 3 | undet | - | 3 | undet | - | 2 | 1.84E+05 | 9.39E+02 |
| 976 | 3 | 1.32E+03 | 3.95E+01 | 3 | 5.00E-01 | 1.92E-02 | 3 | 1.29E+00 | 3.54E-02 | 3 | undet | - | 2 | 3.16E+05 | 1.24E+04 |
| 981 | 3 | 1.93E+04 | 3.00E+02 | 3 | 4.20E-01 | 6.16E-03 | 3 | 6.64E+02 | 1.48E+01 | 2 | 7.27E+05 | 1.96E+03 | 2 | 8.34E+04 | 6.48E+03 |
| 989 | 3 | undet | - | 3 | 1.00E-01 | 9.00E-03 | 3 | 2.20E-01 | 2.36E-02 | 3 | undet | - | 2 | 8.76E+06 | 2.83E+05 |
| 992 | 3 | 1.10E+05 | 5.16E+03 | 3 | 2.12E+03 | 4.44E+01 | 3 | 5.15E+01 | 3.10E+00 | 3 | 2.93E+03 | 9.41E+01 | 2 | 4.24E+06 | 9.04E+04 |
| 994 | 3 | 1.94E+05 | 1.26E+04 | 3 | 3.48E+03 | 8.84E+01 | 3 | 1.36E+03 | 3.85E+03 | 3 | 5.75E+03 | 9.89E+01 | 2 | 1.19E+07 | 9.52E+04 |
| 999 | 3 | 2.25E+04 | 2.64E+02 | 3 | 1.00E-01 | 1.36E-02 | 3 | 3.58E+01 | 8.41E-02 | 3 | undet | - | 2 | 1.28E+06 | 2.08E+04 |
| 1001 | 3 | 5.25E+03 | 2.55E+02 | 3 | 5.88E+00 | 1.68E-01 | 3 | 9.52E+01 | 7.27E+01 | 2 | 5.62E+03 | 2.18E+00 | 2 | 1.17E+06 | 5.73E+04 |
| 1003 | 3 | 1.50E+05 | 1.07E+04 | 3 | 6.29E+03 | 8.38E+01 | 3 | undet | - | 2 | 5.38E+04 | 1.67E+02 | 2 | 1.21E+06 | 1.21E+05 |
| 1009 | 3 | 2.89E+04 | 1.80E+03 | 3 | 3.30E+02 | 1.68E+00 | 3 | 9.83E+02 | 1.13E+01 | 3 | undet | - | 2 | 1.12E+06 | 8.54E+03 |
| 1017 | 3 | 3.06E+04 | 6.94E+02 | 3 | 5.83E+00 | 8.49E-02 | 3 | 1.49E+02 | 5.91E-01 | 2 | 7.05E+03 | 4.55E+01 | 2 | 3.35E+06 | 2.13E+05 |
| 106a | 3 | 8.73E+04 | 5.16E+02 | 3 | 2.52E+01 | 1.01E+00 | 3 | 8.36E+02 | 3.11E+01 | 3 | 2.52E+02 | 1.38E+02 | 2 | 1.23E+07 | 1.35E+05 |
| 119c | 3 | 4.54E+04 | 6.14E+01 | 2 | 2.40E+02 | 4.54E-02 | 2 | 1.12E+04 | 2.41E+00 | 3 | 6.57E+03 | 1.85E+02 | 2 | 9.90E+05 | 6.96E+03 |
| 152e | 3 | 8.06E+04 | 2.73E+03 | 3 | 2.39E+01 | 2.64E-01 | 3 | 9.67E+00 | 4.08E-01 | 3 | 6.71E+03 | 1.44E+02 | 2 | 2.65E+06 | 1.18E+05 |

N, numbers of replicates performed for each sample and quantification test; undet., samples below the detection limi
