# Supplementary material for: Alterations in the Abundance and Co-occurrence of Akkermansia muciniphila and Faecalibacterium prausnitzii in the Colonic Mucosa of Inflammatory Bowel Disease Subjects
Source: Front Cell Infect Microbiol. 2018 Sep 7;8:281. doi: 10.3389/fcimb.2018.00281 (PMC6137959; doi:10.3389/fcimb.2018.00281)
Supplement: Supplementary file 2 [file Table_2.DOCX]

Supplementary Material

**Alterations in the abundance and co-occurrence of *Akkermansia muciniphila* and *Faecalibacterium prausnitzii* in the colonic mucosa of inflammatory bowel disease subjects**

Mireia Lopez-Siles, Núria Enrich-Capó, Xavier Aldeguer, Miriam Sabat-Mir, Sylvia H. Duncan, L. Jesús Garcia-Gil^1^, Margarita Martinez-Medina

*** Correspondence:** L. Jesús Garcia-Gil, [jesus.garcia@udg.edu](mailto:jesus.garcia@udg.edu)

# Supplementary Tables

Table S2. Comparison of original *P-*values and those obtained after FDR analysis. Inconsistencies with original results are indicated in red.

| **Variable** | **overall *P*-value** | **Pairwise comparisons** | **Original *P*-value** | **FDR *P*-value** |
| --- | --- | --- | --- | --- |
| Age | 0.002 | H-IBS | 0.305 | 0.381 |
|  |  | H-UC | 0.165 | 0.236 |
|  |  | H-CD | 0.002 | 0.010 |
|  |  | H-CRC | 0.072 | 0.144 |
|  |  | IBS-UC | 0.594 | 0.660 |
|  |  | IBS-CD | 0.731 | 0.731 |
|  |  | IBS-CRC | 0.100 | 0.167 |
|  |  | UC-CD | 0.067 | 0.144 |
|  |  | UC-CRC | 0.008 | 0.027 |
|  |  | CD-CRC | 0.002 | 0.010 |
| *F.prausnitzii* group of subjects | 0.024 | H-IBS | 0.258 | 0.287 |
|  |  | H-UC | 0.232 | 0.287 |
|  |  | H-CD | 0.021 | 0.140 |
|  |  | H-CRC | 0.028 | 0.140 |
|  |  | IBS-UC | 0.052 | 0.170 |
|  |  | IBS-CD | 0.068 | 0.170 |
|  |  | IBS-CRC | 0.100 | 0.191 |
|  |  | UC-CD | 0.124 | 0.191 |
|  |  | UC-CRC | 0.134 | 0.191 |
|  |  | CD-CRC | 0.688 | 0.688 |
| phylogroup I group of subjects | 0.002 | H-IBS | 0.359 | 0.500 |
|  |  | H-UC | 0.074 | 0.185 |
|  |  | H-CD | <0.001 | <0.001 |
|  |  | H-CRC | 0.008 | 0.040 |
|  |  | IBS-UC | 0.940 | 0.940 |
|  |  | IBS-CD | 0.348 | 0.500 |
|  |  | IBS-CRC | 0.400 | 0.500 |
|  |  | UC-CD | 0.016 | 0.053 |
|  |  | UC-CRC | 0.157 | 0.314 |
|  |  | CD-CRC | 0.731 | 0.812 |
| phylogroup II group of subjects | 0.015 | H-IBS | 0.421 | 0.728 |
|  |  | H-UC | 0.582 | 0.728 |
|  |  | H-CD | 0.052 | 0.260 |
|  |  | H-CRC | 0.793 | 0.881 |
|  |  | IBS-UC | 0.275 | 0.728 |
|  |  | IBS-CD | 0.524 | 0.728 |
|  |  | IBS-CRC | 1.000 | 1.000 |
|  |  | UC-CD | 0.001 | 0.010 |
|  |  | UC-CRC | 0.395 | 0.728 |
|  |  | CD-CRC | 0.486 | 0.728 |
| *F.prausnitzii* CD location | 0.050 | L1-L2 | 0.024 | 0.072 |
|  |  | L1-L3 | 0.088 | 0.132 |
|  |  | L2-L3 | 0.536 | 0.536 |
| phylogroup I CD location | 0.025 | L1-L2 | 0.067 | 0.101 |
|  |  | L1-L3 | 0.010 | 0.030 |
|  |  | L2-L3 | 0.962 | 0.962 |
| *A.muciniphilaCD* age of diagnosis | 0.033 | A1-A2 | 0.006 | 0.018 |
|  |  | A1-A3 | 0.037 | 0.056 |
|  |  | A2-A3 | 0.948 | 0.948 |
| *Akkermansia*: phylogroup I ratio | 0.031 | H-IBS | 0.634 | 0.905 |
|  |  | H-UC | 0.299 | 0.498 |
|  |  | H-CD | 0.001 | 0.013 |
|  |  | H-CRC | 0.244 | 0.498 |
|  |  | IBS-UC | 0.873 | 1.000 |
|  |  | IBS-CD | 0.298 | 0.498 |
|  |  | IBS-CRC | 1.000 | 1.000 |
|  |  | UC-CD | 0.087 | 0.433 |
|  |  | UC-CRC | 1.000 | 1.000 |
|  |  | CD-CRC | 0.271 | 0.498 |
| *Akkermansia*: phylogroup II ratio | 0.017 | H-IBS | 0.090 | 0.228 |
|  |  | H-UC | 0.229 | 0.382 |
|  |  | H-CD | 0.077 | 0.226 |
|  |  | H-CRC | 0.459 | 0.573 |
|  |  | IBS-UC | 0.422 | 0.573 |
|  |  | IBS-CD | 0.028 | 0.142 |
|  |  | IBS-CRC | 1.000 | 1.000 |
|  |  | UC-CD | 0.010 | 0.096 |
|  |  | UC-CRC | 0.810 | 0.900 |
|  |  | CD-CRC | 0.128 | 0.256 |
