# Supplementary material for: Alterations in the Abundance and Co-occurrence of Akkermansia muciniphila and Faecalibacterium prausnitzii in the Colonic Mucosa of Inflammatory Bowel Disease Subjects
Source: Front Cell Infect Microbiol. 2018 Sep 7;8:281. doi: 10.3389/fcimb.2018.00281 (PMC6137959; doi:10.3389/fcimb.2018.00281)
Supplement: Supplementary file 3 [file Image_1.pdf]

Supplementary Material

Alterations in the abundance and co-occurrence of *Akkermansia muciniphila* and *Faecalibacterium prausnitzii* in the colonic mucosa of inflammatory bowel disease subjects

Mireia Lopez-Siles, Núria Enrich-Capó, Xavier Aldeguer, Miriam Sabat-Mir, Sylvia H. Duncan, L. Jesús Garcia-Gil*, Margarita Martinez-Medina

*** Correspondence:** L. Jesús Garcia-Gil, jesus.garcia@udg.edu

Supplementary Figure

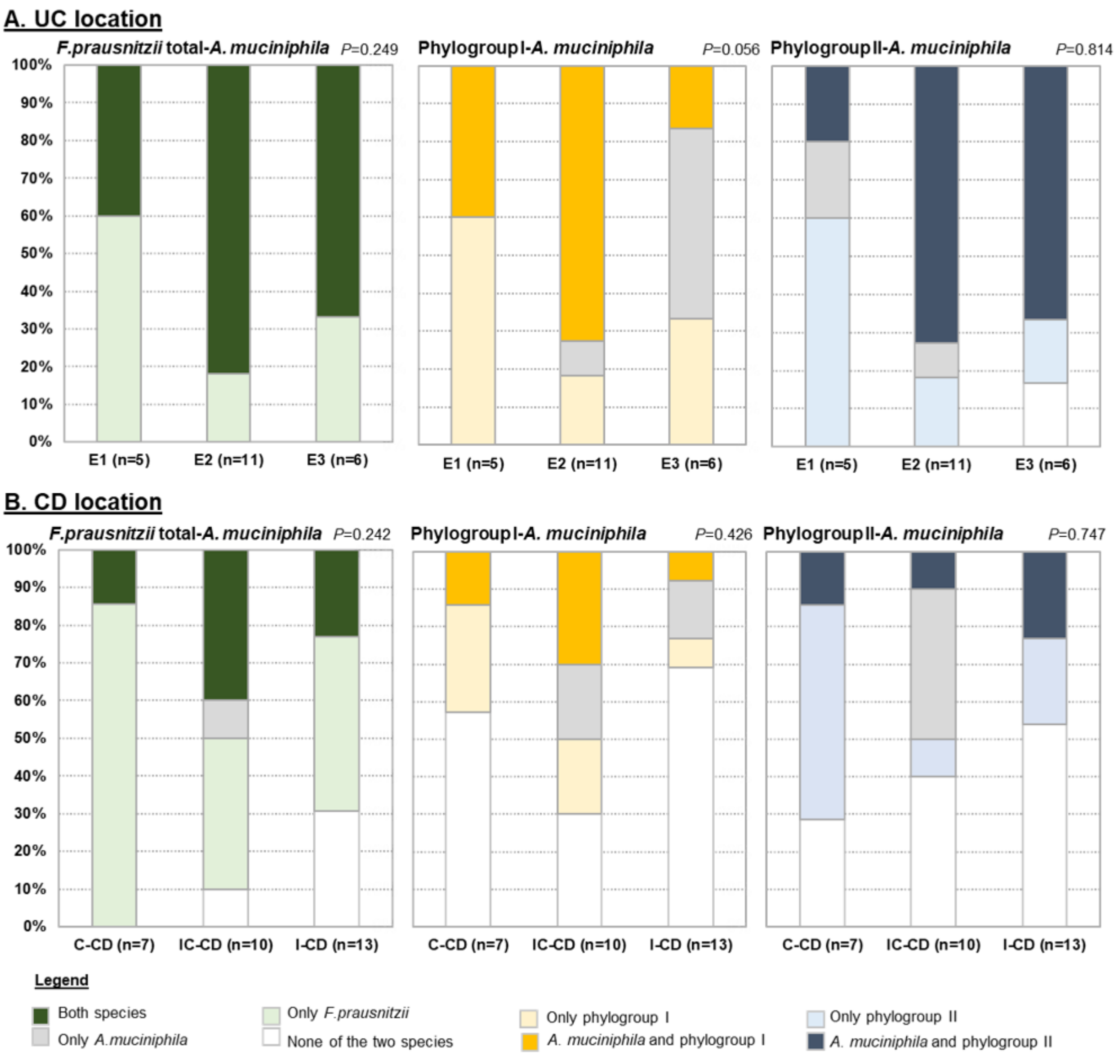

Figure S1. Prevalence of total *F. prausnitzii,* phylogroups and *A. muciniphila* (A) by disease subtype in patients with UC and (B) in patients with CD; UC, ulcerative colitis; CD, Crohn's disease; E1, ulcerative proctitis; E2, ulcerative left-sided colitis; E3, ulcerative pancolitis; IC-CD, ileocolonic CD, I-CD, ileal CD; C-CD, colonic CD.
