# Supplementary material for: Alterations in the Abundance and Co-occurrence of Akkermansia muciniphila and Faecalibacterium prausnitzii in the Colonic Mucosa of Inflammatory Bowel Disease Subjects
Source: Front Cell Infect Microbiol. 2018 Sep 7;8:281. doi: 10.3389/fcimb.2018.00281 (PMC6137959; doi:10.3389/fcimb.2018.00281)
Supplement: Supplementary file 4 [file Image_2.pdf]

Supplementary Material

Alterations in the abundance and co-occurrence of *Akkermansia muciniphila* and *Faecalibacterium prausnitzii* in the colonic mucosa of inflammatory bowel disease subjects

Mireia Lopez-Siles, Núria Enrich-Capó, Xavier Aldeguer, Miriam Sabat-Mir, Sylvia H. Duncan, L. Jesús Garcia-Gil*, Margarita Martinez-Medina

*** Correspondence:** L. Jesús Garcia-Gil, jesus.garcia@udg.edu

Supplementary Figure

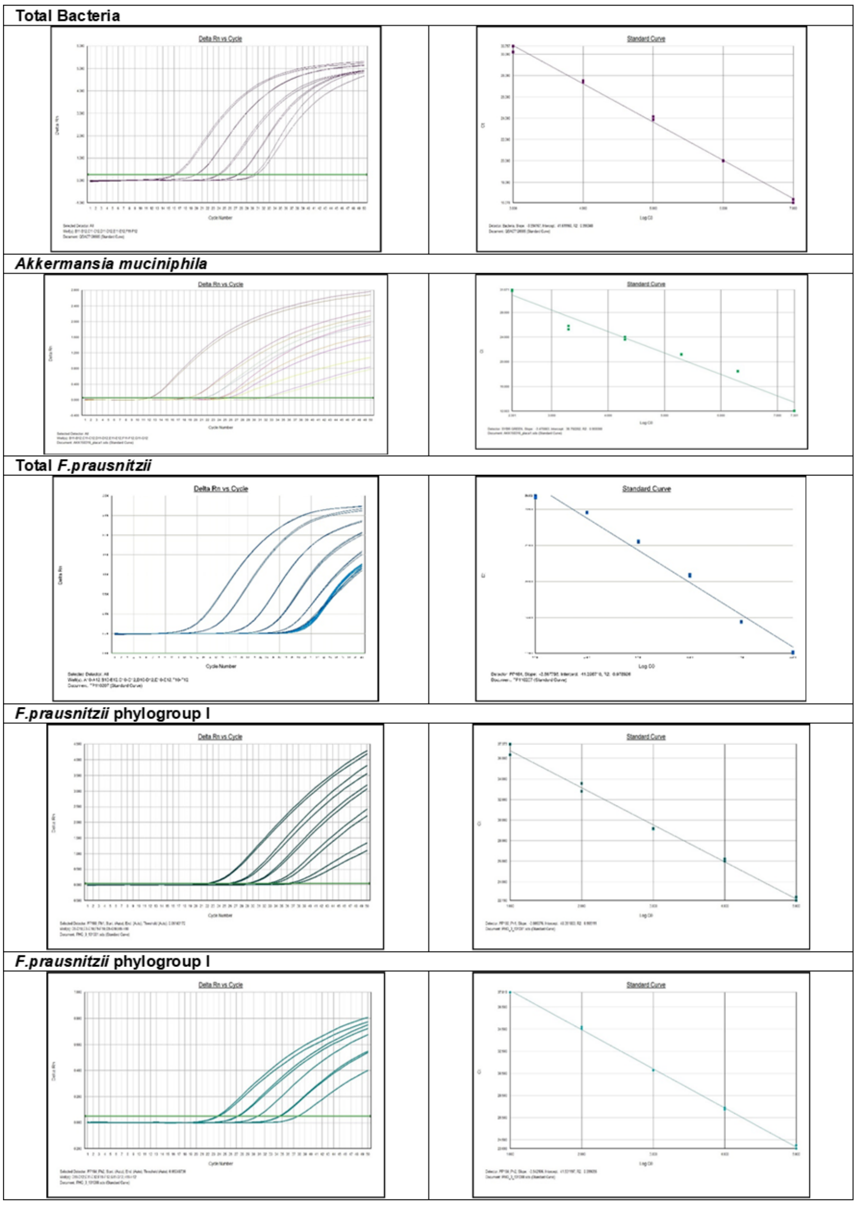

Target microorganism	Calibration curves slope	Calibration curves intercept	PCR efficiency calculated from slope	Amplification factor	R^2 of calibration curve	Dinamic range	LOD
Total Bacteria	-3.594167	41.611160	89.77%	1.90	0.996348	10^7 - 10^3	149.25
Akkermansia muciniphila	-3.475663	38.792202	93.96%	1.94	0.9593	2x10^7 - 2x10^2	1279.38
F. prausnitzii (total)	-3.567798	41.298710	90.67%	1.91	0.978926	10^7 - 10^3	106.67
F. prausnitzii phylogroup I	-3.605976	40.351883	89.37%	1.89	0.995111	10^6 - 10	110.261
F. prausnitzii phylogroup II	-3.542106	41.531197	91.57%	1.92	0.999055	10^6 - 10	239.462

Figure S2. qPCR standard curves and parameters.
