# Supplementary material for: Alterations in the Abundance and Co-occurrence of Akkermansia muciniphila and Faecalibacterium prausnitzii in the Colonic Mucosa of Inflammatory Bowel Disease Subjects
Source: Front Cell Infect Microbiol. 2018 Sep 7;8:281. doi: 10.3389/fcimb.2018.00281 (PMC6137959; doi:10.3389/fcimb.2018.00281)
Supplement: Supplementary file 5 [file Image_3.pdf]

Supplementary Material

Alterations in the abundance and co-occurrence of *Akkermansia muciniphila* and *Faecalibacterium prausnitzii* in the colonic mucosa of inflammatory bowel disease subjects

Mireia Lopez-Siles, Núria Enrich-Capó, Xavier Aldeguer, Miriam Sabat-Mir, Sylvia H. Duncan, L. Jesús Garcia-Gil*, Margarita Martinez-Medina

*** Correspondence:** L. Jesús Garcia-Gil, jesus.garcia@udg.edu

Supplementary Figure

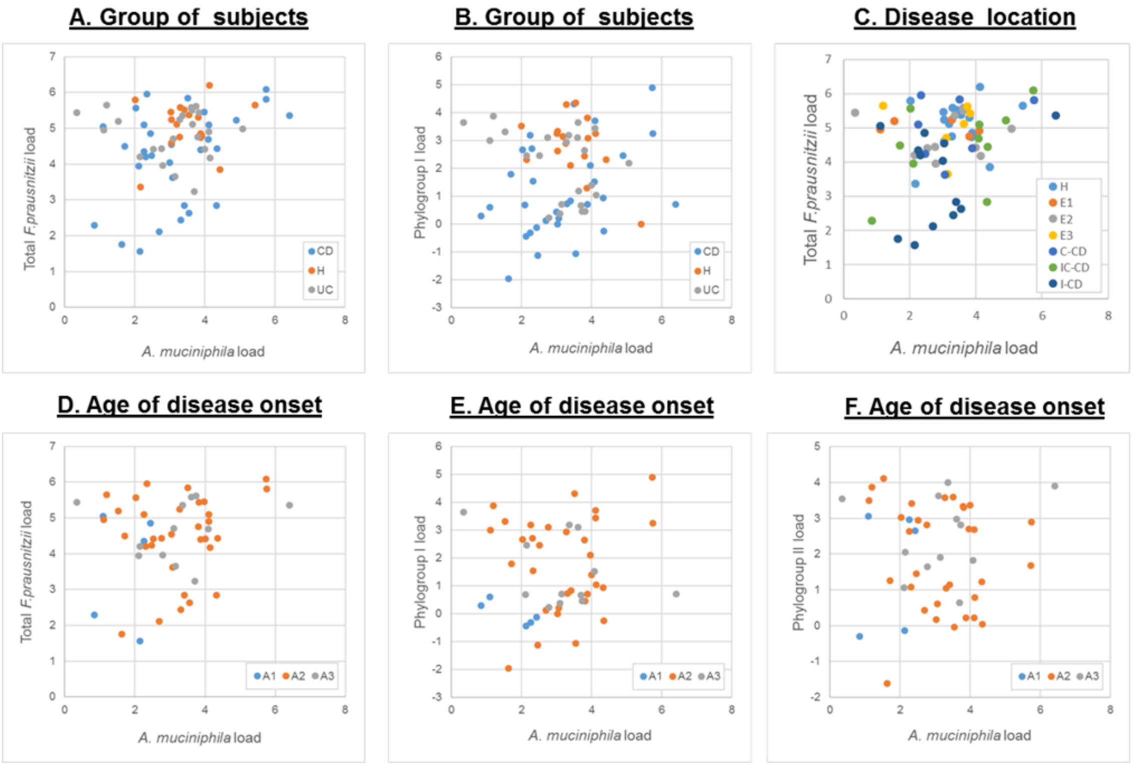

Figure S3. Biplots representing mucosa-associated *F. praunsitzii* and *A.muciniphila* in several groups of subjects (A) Total *F. prausnitzii* and *A muciniphila* load by condition (B) Phylogroup I and *A. muciniphila* by condition. (C) Total *F. prausnitzii* and *A. muciniphila* by inflammatory bowel disease subtype (D to F) Total *F. prausnitzii,* Phylogroup I or Phylogroup II and *A muciniphila* load by age of disease onset, respectively.

H, control subjects; UC, ulcerative colitis; CD, Croh's disease; E1, ulcerative proctitis; E2, ulcerative left-sided colitis; E3, ulcerative pancolitis; IC-CD, ileocolonic CD, I-CD, ileal CD; C-CD, colonic CD.

A. muciniphila load = Log (16S rRNA gene *Akkermansia/*16S rRNA gene million bacteria)

Total *F. prausnitzii* load = Log (16S rRNA gene total *F.prausnitzii*/16S rRNA gene million bacteria)

Phylogroup I load = Log (16S rRNA gene total *F.prausnitzii* phylogroup I/16s rRNA gene million bacteria)

Phylogroup II load = Log (16S rRNA gene total *F.prausnitzii* phylogroup II/16s rRNA gene million bacteria)
